# Relationship between collider bias and interactions on the log-additive scale

**DOI:** 10.1177/09622802241306860

**Published:** 2025-03-02

**Authors:** Apostolos Gkatzionis, Shaun R Seaman, Rachael A Hughes, Kate Tilling

**Affiliations:** 1 MRC Integrative Epidemiology Unit, University of Bristol, Bristol, UK; 2 MRC Biostatistics Unit, University of Cambridge, Cambridge, UK; 3 Department of Population Health Sciences, Bristol Medical School, University of Bristol, Bristol, UK

**Keywords:** collider bias, Berkson’s bias, log-additive model, interaction, inverse probability weighting, avon longitudinal study of parents and children

## Abstract

Collider bias occurs when conditioning on a common effect (collider) of two variables X,Y . In this article, we quantify the collider bias in the estimated association between exposure X and outcome Y induced by selecting on one value of a binary collider S of the exposure and the outcome. In the case of logistic regression, it is known that the magnitude of the collider bias in the exposure–outcome regression coefficient is proportional to the strength of interaction $\delta_{3}$ between X and Y in a log-additive model for the collider: $P(S=1|X,Y)=\exp\;\left\{\delta_{0}+\delta_{1}X+\delta_{2}Y+\delta_{3}XY\right\}$ . We show that this result also holds under a linear or Poisson regression model for the exposure–outcome association. We then illustrate numerically that even if a log-additive model with interactions is not the true model for the collider, the interaction term in such a model is still informative about the magnitude of collider bias. Finally, we discuss the implications of these findings for methods that attempt to adjust for collider bias, such as inverse probability weighting which is often implemented without including interactions between variables in the weighting model.

## 1. Introduction

**Figure 1.**
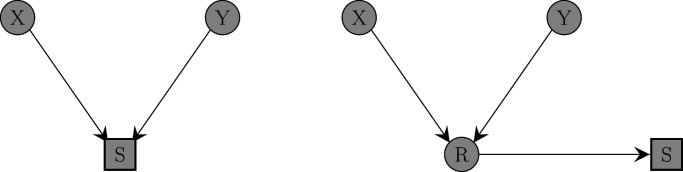
Causal diagram illustrating how collider bias occurs. Two variables X and Y will become correlated when conditioning on a collider (left) or on a variable that is causally downstream of a collider (right), even if they were unconditionally independent. Or, if X and Y are unconditionally associated, conditioning on S will change the strength of their association.

Collider bias is a common concern in epidemiological studies. When exploring the association between an exposure X and an outcome Y of interest, collider bias occurs if the analysis is conditioned on a common effect of the exposure and outcome, or a variable that is causally downstream of the common effect (a “child” of the collider), as illustrated in Figure 1. Numerous examples of studies affected by collider bias can be found in the literature. For example, collider bias has been suggested as an explanation for the “obesity paradox,” where obesity often appears to be associated with decreased mortality in older individuals or people suffering from chronic diseases, despite being associated with increased mortality in the overall population.^1,2^

In this article, we focus on collider bias induced when an analysis is restricted to a single level of a binary collider variable S . A number of biases in epidemiological studies across a wide range of study designs can be attributed to this mechanism. This includes some forms of selection bias due to non-representative sampling,^3^ survival bias, Berkson’s hospitalization bias,^4^ and index event bias in studies of disease progression,^5^ among others. In all of these cases, the collider S represents the selection into the study: individuals with S=1 have their exposure and outcome observed, while individuals with S=0 do not and are hence excluded from the study.

It has long been recognized in the literature that this type of collider bias relates to interactions between the exposure and the outcome on the log-additive scale in their effects on the collider.^6,7^ Consider the following log-additive model for the collider:

(1) $$\log\;P(S=1|X,Y)=\delta_{0}+\delta_{1}X+\delta_{2}Y+\delta_{3}XY$$

The parameter $\delta_{3}$ quantifies the strength of the exposure–outcome interaction. In analyses of binary outcomes, a number of authors have suggested that collider bias will not affect estimates of the exposure–outcome odds ratio when X and Y do not interact in their effects on S , that is, when $\delta_{3}=0$ .^8–14^ An overview of the relevant literature can be found by Jiang and Ding,^15^ who also explored the direction in which collider bias acts, while Mansournia et al.^16^ obtained similar results and also investigated bias when conditioning on S=0 . Despite the interest this topic has attracted, most of the relevant papers have focused on binary outcome variables, with some papers also restricting the exposure to be a binary variable. One exception is Shahar and Shahar,^17^ who considered a discrete exposure and a discrete outcome, potentially with more than two categories.

In this article, we expand the literature by considering a wider range of outcome variables, including count outcomes (Poisson regression) and continuous outcomes (linear regression) in addition to binary ones. We also place no conditions on the form of the exposure variable. We show that collider bias will not affect the exposure–outcome association when the exposure and outcome do not interact in their effects on the collider, that is, when $\delta_{3}=0$ in model (1). When they do interact, we show that the magnitude of collider bias induced in the exposure–outcome regression coefficients in linear, logistic, and Poisson regression models is proportional to $\delta_{3}$ . Finally, when model (1) is misspecified, we show numerically that for binary X there is still a linear relationship between the magnitude of the collider bias and the estimated value of $\delta_{3}$ in the (misspecified) model (1).

These results have important implications for methods attempting to adjust for collider bias, such as inverse probability weighting (IPW) (Seaman and White^18^). Implementing IPW requires the specification of a model for the collider S . A wide range of statistical models can be used for this task,^19–23^ but in practice applied researchers often use a logistic regression model without interactions, for simplicity. In this article, we show that collider bias depends crucially on variable interactions in the model for S . Therefore, if IPW is implemented using a simple logistic weighting model that does not include interactions, it will not adequately adjust for collider bias.

The rest of this article is organized as follows. In Section 2, we review the relevant literature. We then consider regression models for a binary outcome Y (primarily logistic regression), linear regression, and Poisson regression. For each of these models, we prove that the magnitude of collider bias is proportional to the value of the exposure–outcome interaction $\delta_{3}$ in the log-additive model (1). In Section 3, we investigate collider bias when the collider variable is not distributed according to the log-additive model (1). We demonstrate numerically that when X is a binary variable, estimating $\delta_{3}$ can still provide information about the magnitude of collider bias, even if model (1) is not the true model for S . Section 4 contains an illustrative application using data from the Avon Longitudinal Study of Parents and Children (ALSPAC) to investigate associations between maternal traits, such as education or smoking before pregnancy, and offspring sex. A summary of our main findings and their implications for applied analyses is presented in Section 5.

## 2. Collider bias under a log-additive model for the collider

### 2.1. Statement of the problem

As in Section 1, suppose that the objective is to investigate the association between an exposure X and an outcome Y . We focus on the marginal association between X and Y . However, the results presented here can be extended to cover the conditional association between X and Y given a set of other variables, for example, confounders, as we briefly discuss towards the end of Section 2.2.1.

As in the previous section, we let the collider S represent a binary selection indicator. The unconditional exposure–outcome association cannot be estimated directly using the observed data; instead, only the conditional association given S=1 can be estimated. As stated in Section 1, if an individual’s exposure and outcome values affect their likelihood of selection into the study sample, the conditional and unconditional exposure–outcome associations will differ. Our aim in this section is to explore the difference between the conditional and unconditional exposure–outcome associations for a binary collider variable distributed according to model (1).

We study collider bias separately for binary, continuous and count outcome variables. For binary outcome variables, we examine collider bias in odds ratios, logistic regression coefficients and risk ratios. For continuous outcomes, we quantify collider bias in linear regression coefficients, and for count outcome variables, we investigate collider bias in the log rate ratio parameters of a Poisson regression model. We work under the assumption that the collider S is distributed according to the log-additive model (1); different models for S will be considered in the next section.

### 2.2. The relationship between collider bias and exposure–outcome interactions

#### 2.2.1. Binary outcome—Collider bias on the odds ratio scale

Consider first the case of a binary outcome, which has received the most attention in the literature (e.g. Bartlett et al.^8^ and Jiang and Ding^15^). Some of these papers also restricted the exposure to be binary; here, we do not place any assumptions on the type of the exposure variable. Our main assumption is that the exposure and outcome affect the collider on the log-additive scale, as in (1). Let

$$\begin{array}{rl} OR_{XY}(x) & =\frac{P(Y=1|X=x+1)}{P(Y=0|X=x+1)}\times \frac{P(Y=0|X=x)}{P(Y=1|X=x)}\\ OR_{XY|S=1}(x) & =\frac{P(Y=1|X=x+1,S=1)}{P(Y=0|X=x+1,S=1)}\times \frac{P(Y=0|X=x,S=1)}{P(Y=1|X=x,S=1)} \end{array}$$

be the unconditional and conditional odds ratios, respectively.

In Supplemental Section 2.1 (see also Jiang and Ding^15^), we prove that

(2) $$OR_{XY|S=1}(x)=OR_{XY}(x)\;\exp\;\left\{\delta_{3}\right\}$$

This shows that the magnitude of collider bias on the odds ratio scale is fully determined by the interaction parameter $\delta_{3}$ . In particular, if $\delta_{3}=0$ , we have $OR_{XY|S=1}(x)=OR_{XY}(x)$ , meaning that collider bias does not occur. It follows from equation (2) that if the logistic model

(3) $$\text{logit}P(Y=1|X)=\beta_{0}+\beta_{1}X$$

is correctly specified, the magnitude of collider bias in the parameter $\beta_{1}$ is equal to $\delta_{3}$ . Letting $\beta_{1}^{S}$ denote the (population) log-odds ratio for the exposure–outcome association conditional on S=1 , one could write

(4) $$\beta_{1}^{S}=\beta_{1}+\delta_{3}$$

Some generalizations of this result are possible. For example, equation (4) still holds when the X−S and Y−S main effects in model (1) are replaced by non-linear functions:

$$\log\;P(S=1|X,Y)=g_{1}(X)+g_{2}(Y)+\delta_{3}XY$$

In addition, consider the more general case of a higher-order (in *X*) interaction:

$$\log\;P(S=1|X,Y)=g_{1}(X)+g_{2}(Y)+g_{3}(X)Y$$

In this case, equation (2) becomes

$$OR_{XY|S=1}(x)=OR_{XY}(x)\exp\;\left\{g_{3}(x+1)-g_{3}(x)\right\}$$

The pattern of bias will, therefore, depend on the form of the function $g_{3}$ . Note that the conditional and unconditional odds ratios will be equal if and only if $g_{3}(x+1)=g_{3}(x)$ for all x , i.e. $g_{3}$ is constant in X , which again shows that collider bias occurs if and only if the exposure and outcome interact in their effects on the collider.

Finally, let the variable X be vector-valued; this may represent either multiple exposures whose association with Y is investigated, or a single exposure whose association with the outcome is adjusted for the presence of observed confounders. Under models (1) and (3), where now $\beta_{1},\delta_{1},\mathrm{and}\delta_{3}$ are vector-valued, one can show that $\beta_{1}^{S}=\beta_{1}+\delta_{3}$ , that is, the bias in the regression coefficient of an element $X_{j}$ of the vector X is equal to the interaction between $X_{j}$ and Y in the collider model. Moreover, interactions between the variables $X_{j}$ in the collider model will not affect the bias.

#### 2.2.2. Binary outcome—Collider bias on the risk ratio scale

We now explore the magnitude of collider bias on the risk ratio scale. Once again, we assume that the collider S is distributed according to (1) and that the outcome Y is binary. Our aim is to compare the unconditional risk ratio

$$RR_{XY}(x)=\frac{P(Y=1|X=x+1)}{P(Y=1|X=x)}$$

to the conditional risk ratio

$$RR_{XY|S=1}(x)=\frac{P(Y=1|X=x+1,S=1)}{P(Y=1|X=x,S=1)}$$

In Supplemental Section 2.2, we prove that

(5) $$RR_{XY|S=1}(x)=RR_{XY}(x)\times \frac{e^{\delta_{2}+\delta_{3}(x+1)}\;P(Y=1|X=x)+e^{\delta_{3}}\;P(Y=0|X=x)}{e^{\delta_{2}+\delta_{3}(x+1)}\;P(Y=1|X=x+1)+P(Y=0|X=x+1)}$$

It is clear from this formulation that the properties we have proved for the magnitude of collider bias on the odds ratio scale do not hold for bias on the risk ratio scale; for example, unlike (4), expression (5) involves both the interaction parameter $\delta_{3}$ and the outcome-collider coefficient $\delta_{2}$ . A more specific formula for the bias on the risk ratio scale can be obtained by incorporating modeling assumptions for the exposure–outcome relationship into (5). In practice, risk ratios are often studied under the log-binomial regression model

$$\text{log}P(Y=1|X=x)=\beta_{0}+\beta_{1}x$$

This implies an unconditional risk ratio of $RR_{XY}(x)=e^{\beta_{1}}$ for any x , and from (5), a conditional risk ratio of

$$RR_{XY|S=1}(x)=\frac{e^{\delta_{2}+\delta_{3}(x+1)}\;e^{\beta_{0}+\beta_{1}(x+1)}+e^{\delta_{3}+\beta_{1}}\;(1-e^{\beta_{0}+\beta_{1}x})}{e^{\delta_{2}+\delta_{3}(x+1)}\;e^{\beta_{0}+\beta_{1}(x+1)}+(1-e^{\beta_{0}+\beta_{1}(x+1)})}$$

The difference between the unconditional and conditional risk ratios is then

(6) $$RR_{XY}(x)-RR_{XY|S=1}(x)=e^{\beta_{1}}\;\left(1-\frac{e^{\delta_{2}+\delta_{3}(x+1)}\;e^{\beta_{0}+\beta_{1}x}+e^{\delta_{3}}\;(1-e^{\beta_{0}+\beta_{1}x})}{e^{\delta_{2}+\delta_{3}(x+1)}\;e^{\beta_{0}+\beta_{1}(x+1)}+(1-e^{\beta_{0}+\beta_{1}(x+1)})}\right)$$

The bias hence depends on both the interaction term $\delta_{3}$ and the outcome-collider parameter $\delta_{2}$ , and the absence of an interaction ( $\delta_{3}=0$ ) is not enough to eliminate bias on the risk ratio scale. On the other hand, one can easily verify that the conditional and unconditional risk ratios are equal when $\delta_{2}=\delta_{3}=0$ , that is, when the outcome does not associate with the collider; and likewise, there is no bias if $\delta_{3}=0$ and $\beta_{1}=0$ .

In Supplemental Section 2.2, we explore collider bias on the risk ratio scale under a logistic regression model for the outcome and obtain results similar to those reported here.

#### 2.2.3. Continuous outcome—Collider bias in linear regression coefficients

We now turn our attention to continuous outcome variables, and assume that the outcome is distributed according to the linear regression model

$$Y=\beta_{0}+\beta_{1}X+\epsilon_{Y}$$

where $\epsilon_{Y}∼N(0,\sigma^{2})$ independent of X . We start by noting that $E(Y|X)=\beta_{0}+\beta_{1}X$ and explore the bias in the regression coefficient $\beta_{1}$ when conditioning on S=1 . In Supplemental Section 2.3, we show that the conditional expectation E(Y|X,S=1) is equal to

(7) $$E(Y|X,S=1)=(\beta_{0}+\delta_{2}\sigma^{2})+(\beta_{1}+\delta_{3}\sigma^{2})x$$

Denoting by $\beta_{0}^{S}$ and $\beta_{1}^{S}$ the regression coefficients of a linear regression model conditioned on S=1 , it follows that

(8) $$\beta_{1}^{S}=\beta_{1}+\delta_{3}\sigma^{2}$$

which implies that the two regression coefficients in the conditional and unconditional exposure–outcome models will be equal if and only if $\delta_{3}=0$ . When the two coefficients differ, the magnitude of collider bias induced is equal to the interaction term $\delta_{3}$ multiplied by the residual variance $\sigma^{2}$ . Moreover, $\beta_{0}^{S}=\beta_{0}+\delta_{2}\sigma^{2}$ ; therefore, the bias in the intercept $\beta_{0}$ is equal to the outcome-collider parameter $\delta_{2}$ multiplied by the residual variance.

As with binary outcomes, the above derivation allows for a non-linear exposure-collider effect,

$$\log\;P(S=1|X,Y)=g_{1}(X)+\delta_{2}Y+\delta_{3}XY$$

but not for a non-linear outcome-collider effect. Finally, a more general regression framework for the exposure–outcome relationship can be considered as follows:

$$Y=m(X;\beta )+\epsilon ,\;\epsilon ∼N(0,\sigma^{2})$$

where m(x;β)=E(Y|X=x) is a potentially non-linear function that represents the exposure–outcome association. This yields

$$E(Y|X=x,S=1)=m(x;\beta )+\sigma^{2}\delta_{2}+\sigma^{2}\delta_{3}x$$

In addition, as for logistic regression, our results still hold if X is vector-valued.

#### 2.2.4. Count outcome—Collider bias in poisson regression coefficients

Finally, we consider the case of a count outcome variable distributed according to the Poisson regression model

(9) $$Y|X=x∼Poisson(\lambda ),\;\lambda =\lambda (x)=\exp\;\left\{\beta_{0}+\beta_{1}x\right\}$$

Once again, our aim is to obtain an expression for the bias in the regression coefficient $\beta_{1}$ when the collider S follows the log-additive model (1). Our framework here has some similarities to the work of Shahar and Shahar^17^; their paper requires that the exposure is a discrete variable but does not place any distributional assumptions on the outcome, apart from it being discrete.

In Supplemental Section 2.4, we show that if expression (9) holds, then Y|X,S=1 is Poisson( κ )-distributed, where

(10) $$\kappa =\kappa (X)=\exp\;\left\{(\beta_{0}+\delta_{2})+(\beta_{1}+\delta_{3})X\right\}$$

Therefore, the relationship between the regression coefficients $\beta_{0}$ and $\beta_{1}$ in the unconditional exposure–outcome model and the corresponding coefficients $\beta_{0}^{S}$ and $\beta_{1}^{S}$ in the conditional model is

(11) $$\begin{array}{rl} \beta_{0}^{S} & =\beta_{0}+\delta_{2}\\ \beta_{1}^{S} & =\beta_{1}+\delta_{3} \end{array}$$

As in the case of logistic regression (4), this implies that the magnitude of collider bias in the regression coefficient $\beta_{1}$ induced by conditioning on S=1 is equal to $\delta_{3}$ . In particular, when the exposure and outcome do not interact in their effects on the collider in the log-additive model (1), that is, $\delta_{3}=0$ , there is no bias.

As with binary and continuous outcome variables, a few extensions of this result are possible, including to analyses with a non-linear exposure-collider association: if expression (9) holds and

$$\log\;P(S=1|X,Y)=g_{1}(X)+\delta_{2}Y+g_{3}(X)Y$$

then Y|X,S=1∼Poisson(κ(x)) , where $\kappa (x)=\exp\;\{\beta_{0}+\beta_{1}x+\delta_{2}+g_{3}(x)\}$ . Finally, our results can be readily extended to Poisson regression with a vector-valued exposure variable.

## 3. Collider bias under alternative models for the collider

### 3.1. Study design

So far we have assumed that the collider S is distributed according to the log-additive model (1). Under this assumption, we have shown that there is a linear relationship between the magnitude of collider bias and the interaction term $\delta_{3}$ in model (1), as shown in equations (4), (8), and (11). However, the log-additive model (1) may be misspecified. In this section, we investigate collider bias under misspecification of model (1). Focusing on the case of a binary exposure variable, we demonstrate two things. First, if the true model for S is not log-additive, the exposure–outcome interaction term in that model does not exhibit a linear relationship with the magnitude of collider bias in exposure–outcome regression coefficients. Second, the limiting value (as the sample size tends to infinity) of the maximum likelihood estimator (MLE) of the interaction parameter $\delta_{3}$ obtained by fitting the log-additive model (1) for S still exhibits a linear relationship with the magnitude of collider bias, even if this log-additive model is misspecified.

Our analysis here is asymptotic in nature: the aim is to obtain asymptotic results about the relationship between the limiting value of the MLE of $\delta_{3}$ and collider bias. We have not proved such results analytically when model (1) is misspecified; instead, we calculate each limiting value by generating a very large dataset and evaluating the MLE on this dataset (i.e. equivalent to calculating the expected score function using Monte Carlo integration, setting this expectation equal to zero and solving the score equations). To reflect that, we will refer to our analysis as a “numerical asymptotic study.”

We considered nine data generating mechanisms, obtained by combining three different outcome models and three different models for the selection indicator S . For the outcome, we used linear, logistic, and Poisson regressions:

$$\begin{array}{rl} \left(Y_{1}\right) & :\text{logit}P(Y=1|X)=\beta_{0}^{Y_{1}}+\beta_{1}^{Y_{1}}X\\ \left(Y_{2}\right) & :Y=\beta_{0}^{Y_{2}}+\beta_{1}^{Y_{2}}X+\epsilon_{Y},\;\epsilon_{Y}∼N(0,\sigma^{2})\\ \left(Y_{3}\right) & :Y|X=x∼\mathrm{Poisson}(\lambda ),\;\lambda =\lambda (x)=\exp\;\left\{\beta_{0}^{Y_{3}}+\beta_{1}^{Y_{3}}x\right\} \end{array}$$

For the selection indicator S , we considered standard logistic and probit regression models:

$$\begin{array}{rl} \left(S_{1}\right) & :P(S=1|X,Y)\;=\text{expit}\left\{\delta_{0}^{S_{1}}+\delta_{1}^{S_{1}}X+\delta_{2}^{S_{1}}Y+\delta_{3}^{S_{1}}XY\right\}\\ \left(S_{2}\right) & :S\;=1_{S^{\prime }>0},\;S^{\prime }∼N\left(\delta_{0}^{S_{2}}+\delta_{1}^{S_{2}}X+\delta_{2}^{S_{2}}Y+\delta_{3}^{S_{2}}XY,1.6^{2}\right) \end{array}$$

We also considered a third model, where we generated a latent, normally distributed variable $S^{\prime }$ and then set S=1 for individuals for which the latent variable took values below a lower threshold $r_{1}$ or above an upper threshold $r_{2}$ :

$$(S_{3}):S=1_{S^{\prime }>r_{1}\;\text{or}\;S^{\prime }<r_{2}},\;S^{\prime }∼N\left(\delta_{0}^{S_{3}}+\delta_{1}^{S_{3}}X+\delta_{2}^{S_{3}}Y+\delta_{3}^{S_{3}}XY,1.6^{2}\right)$$

We will refer to ( $S_{3}$ ) as a “double-threshold” model. Finally, in all nine data generating mechanisms, the exposure values were generated from a Bernoulli(0.3) distribution.

The parameters of the three outcome models and the three data-generating models for S were specified as follows. In the outcome model, we set $\beta_{0}^{Y_{j}}=0$ and $\beta_{1}^{Y_{j}}=0.2$ , j=1,2,3 . In the linear regression model ( $Y_{2}$ ), we also set σ=0.5 . In the model for S , the exposure-collider and outcome-collider association parameters were set to $\delta_{1}^{S_{k}}=\delta_{2}^{S_{k}}=0.3$ , k=1,2,3 . Finally, the residual variance for the latent variable $S^{\prime }$ in models ( $S_{2}$ ) and ( $S_{3}$ ) was set to 1.6 so that the regression coefficients in these models represented a comparable strength of association as the coefficients of the logistic regression model ( $S_{1}$ ) (Wooldridge,^24^ Chapter 17).

There were two parameters to be varied in our numerical asymptotic study: the strength of the exposure–outcome interaction $\delta_{3}^{S_{k}}$ , and the proportion of selected individuals, which was determined by the intercept term $\delta_{0}^{S_{k}}$ in the model for S (and for model ( $S_{3}$ ), by the thresholds $r_{1},r_{2}$ ). We conducted two experiments varying the values of these parameters. In the first experiment, we specified the intercept term $\delta_{0}^{S_{k}}$ so that ∼50% of individuals were included in the conditional analysis ( S=1 ); in model ( $S_{3}$ ), we instead set $\delta_{0}^{S_{3}}=0$ and set $r_{1},r_{2}$ equal to the first and third quartiles of the distribution of $S^{\prime }$ . We then varied the exposure–outcome interaction parameter $\delta_{3}^{S_{k}}$ , letting it take the values $\delta_{3}^{S_{k}}=0,\pm 0.1,\pm 0.2,\pm 0.3,\pm 0.4,\mathrm{and}\pm 0.5$ . In our second experiment, we used the same range of values for $\delta_{3}^{S_{k}}$ but varied the value of the intercept $\delta_{0}^{S_{k}}$ . We specified four values for $\delta_{0}^{S_{k}}$ , such that the proportion of selected individuals was 10% , 30% , 70% , and 90% , respectively. For model ( $S_{3}$ ), we set $\delta_{0}^{S_{3}}=0$ and specified the proportion of selected individuals by tuning the thresholds $r_{1},r_{2}$ instead.

For each of the nine data generating mechanisms and each set of parameter values, we generated a single dataset of size $n=10^{7}$ . We used a large sample size to approximate an infinite sample: the values of MLEs obtained using our sample will be very close to the limiting value of these estimators. We then fitted model ( $Y_{j}$ ) using only data on individuals with S=1 to calculate the estimate ${\hat{\beta }}_{1}^{Y_{j}}$ of $\beta_{1}^{Y_{j}}$ . The difference between this estimate and the true value of $\beta_{1}^{Y_{j}}$ is the collider bias induced in the regression coefficient of model ( $Y_{j}$ ). This bias was plotted against the value of the interaction parameter $\delta_{3}^{S_{k}}$ in the correctly specified model ( $S_{k}$ ), k=1,2,3 , to assess their relationship. We then fitted the misspecified log-additive model:

$$(S_{0})\;:\;P(S=1|X,Y)=\exp\;\left\{\delta_{0}^{S_{0}}+\delta_{1}^{S_{0}}X+\delta_{2}^{S_{0}}Y+\delta_{3}^{S_{0}}XY\right\}$$

and computed the MLE ${\hat{\delta }}_{3}^{S_{0}}$ of the interaction parameter $\delta_{3}^{S_{0}}$ , and then plotted this estimate against the collider bias. The estimate ${\hat{\delta }}_{3}^{S_{0}}$ was used in our numerical asymptotic study to approximate the limiting value ${\tilde{\delta }}_{3}^{S_{0}}$ of the MLE; note that ${\tilde{\delta }}_{3}^{S_{0}}$ is the value that minimizes the Kullback-Leibler divergence between model ( $S_{0}$ ) and the true data-generating model for S (which here is model ( $S_{1}$ ), ( $S_{2}$ ), or ( $S_{3}$ )).

Data were generated and models were fitted using R. The log-additive models were fitted as Poisson regression models, using the glm function.

**Figure 2.**
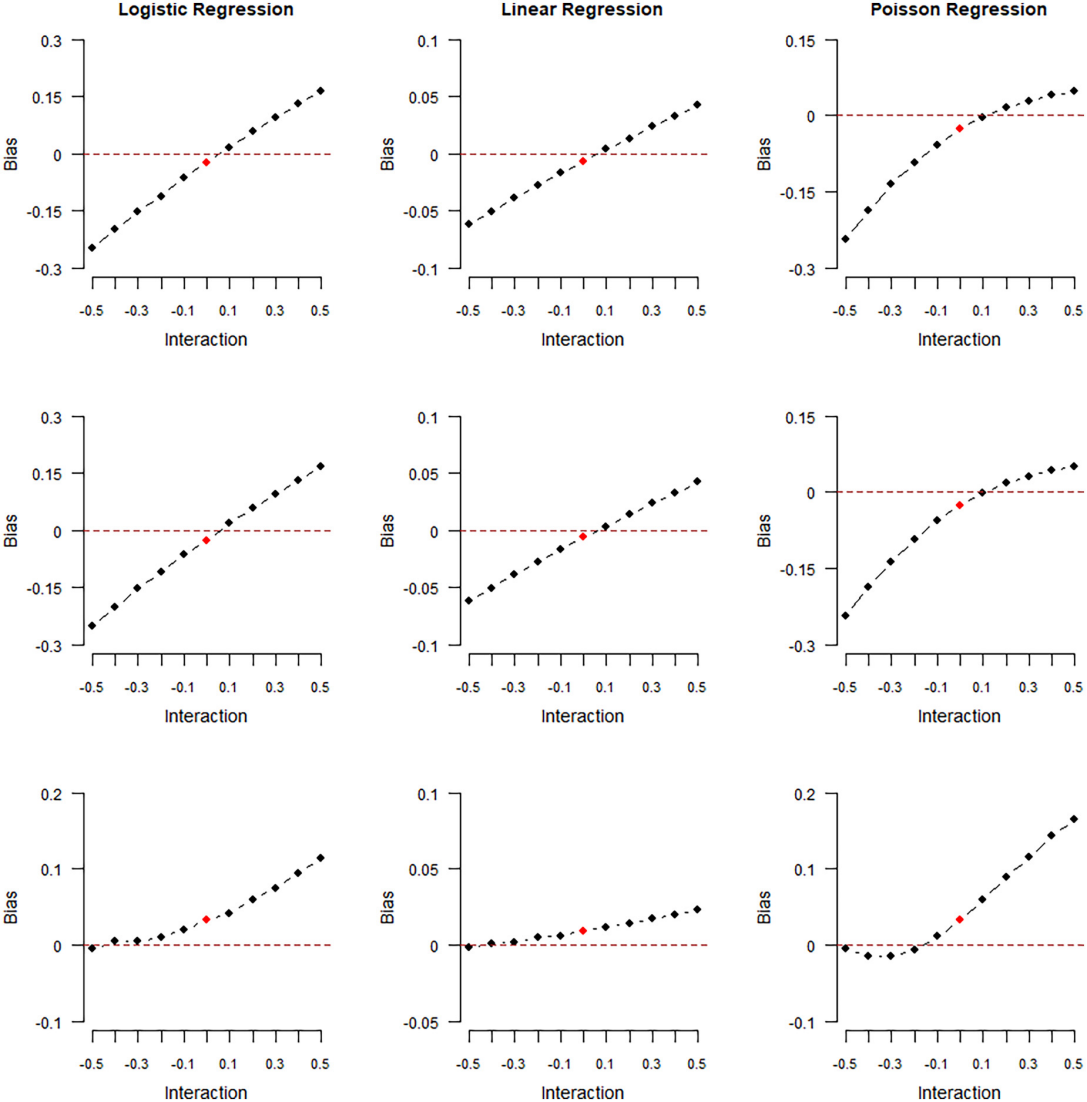
Magnitude of collider bias induced in the exposure–outcome regression coefficient by restricting the analysis to selected ( S=1 ) individuals. Data were generated for $n=10^{7}$ individuals, and the average selection probability was 50% . Outcome data were generated from logistic regression (left column), linear regression (middle column), or Poisson regression (right column) and collider values were generated from logistic regression (model $S_{1}$ , top row), probit regression (model $S_{2}$ , middle row), or the “double threshold” model (model $S_{3}$ , bottom row). The bias is plotted against the exposure–outcome interaction $\delta_{3}^{S_{k}}$ in the collider model. Red color represents the scenario where the logistic model generating S did not contain an interaction between X and Y .

### 3.2. Results

**Figure 3.**
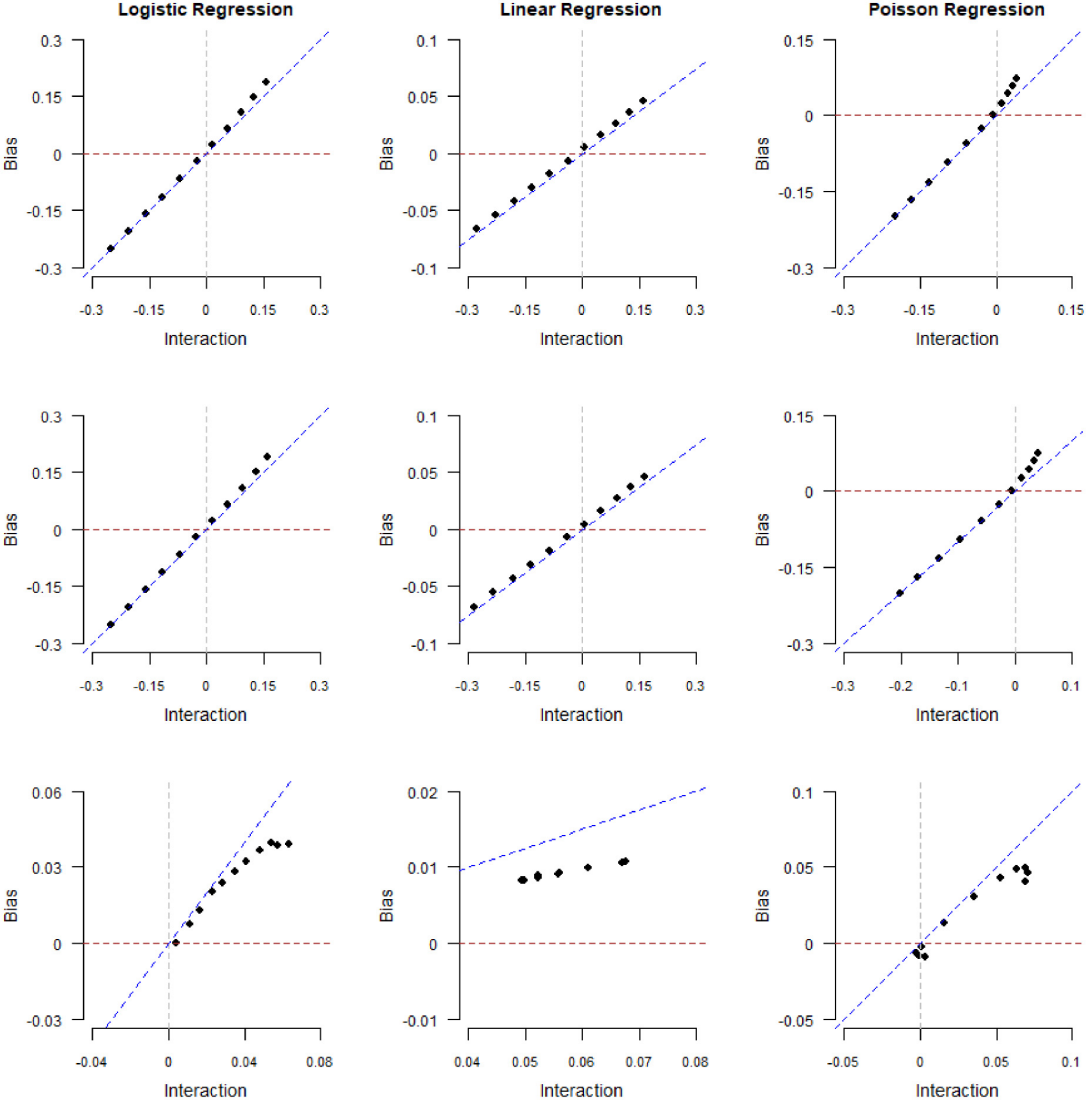
Magnitude of collider bias induced in the exposure–outcome regression coefficient by restricting the analysis to selected ( S=1 ) individuals. Data were generated for $n=10^{7}$ individuals, and the average selection probability was 50% . Outcome data were generated from logistic regression (left column), linear regression (middle column), or Poisson regression (right column) and collider values were generated from logistic regression (model $S_{1}$ , top row), probit regression (model $S_{2}$ , middle row) or the “double threshold” model (model $S_{3}$ , bottom row). The bias is plotted against the estimated values of the exposure–outcome interaction parameter $\delta_{3}^{S_{0}}$ in a (misspecified) log-additive model for S . A gray vertical line represents no interaction ( ${\hat{\delta }}_{3}^{S_{0}}=0$ ).

The results of our first numerical experiment are shown in Figures 2 and 3 and reported in the tables in Supplemental Section 3. In the plots of Figure 2, the collider S was generated under a logistic (top row of plots), probit (middle row), or “double-threshold” (bottom row) model, while the outcome was generated from a logistic (left column), linear (centre column), or Poisson (right column) regression model. The collider bias is plotted against the true value of the interaction parameter $\delta_{3}^{S_{k}}$ in the corresponding collider model ( $S_{k}$ ), k=1,2,3 . The relationship between collider bias and the values of the interaction parameters is not linear, with deviations from linearity being more pronounced for Poisson regression and less so for linear regression. In addition, the collider bias is quite small in scenarios where the exposure and outcome do not interact in their effects on the collider ( $\delta_{3}^{S_{k}}=0$ , plotted in red), although even here some bias still exists.

For some of the models considered here, it is possible to derive analytic expressions for collider bias using arguments similar to those in the previous section. As an example, in Supplemental Section 2.5, we obtain an expression for the collider bias in the exposure–outcome regression coefficient of a logistic regression model when the collider S also follows a logistic regression model. However, such relationships can only be derived for relatively simple models, and the bias will generally depend on all the parameters of the collider model, not just on the interaction term.

In Figure 3, we plot collider bias against the limiting values ${\tilde{\delta }}_{3}^{S_{0}}$ of the log-additive interaction parameter $\delta_{3}^{S_{0}}$ , estimated by fitting the misspecified log-additive model ( $S_{0}$ ). Note that the points in these plots are not equally spaced along the *x*-axis because equally spaced interactions on the scale of models ( $S_{1}$ )–( $S_{3}$ ) do not correspond to equally spaced interactions on the log-additive scale. The relationship between collider bias and limiting values of the estimator of $\delta_{3}^{S_{0}}$ appears to be linear, with a slope of 1 for logistic and Poisson regression and $\sigma^{2}=0.25$ in the case of linear regression. This is the same relationship suggested by our theory (equations (4), (8), and (11)), despite the fact that the log-additive model is misspecified.

Note that the linear pattern of collider bias presented in Figure 3 only occurs when the exposure X is binary. In Supplemental Section 4, we report results for a normally distributed exposure variable, where the relationship between bias and ${\tilde{\delta }}_{3}^{S_{0}}$ is not linear (see also Campbell et al.^25^). Unlike the theoretical results in Section 2 of our article, the distribution of the exposure can affect the magnitude of collider bias when the collider S is not generated from the log-additive model ( $S_{0}$ ).

**Figure 4.**
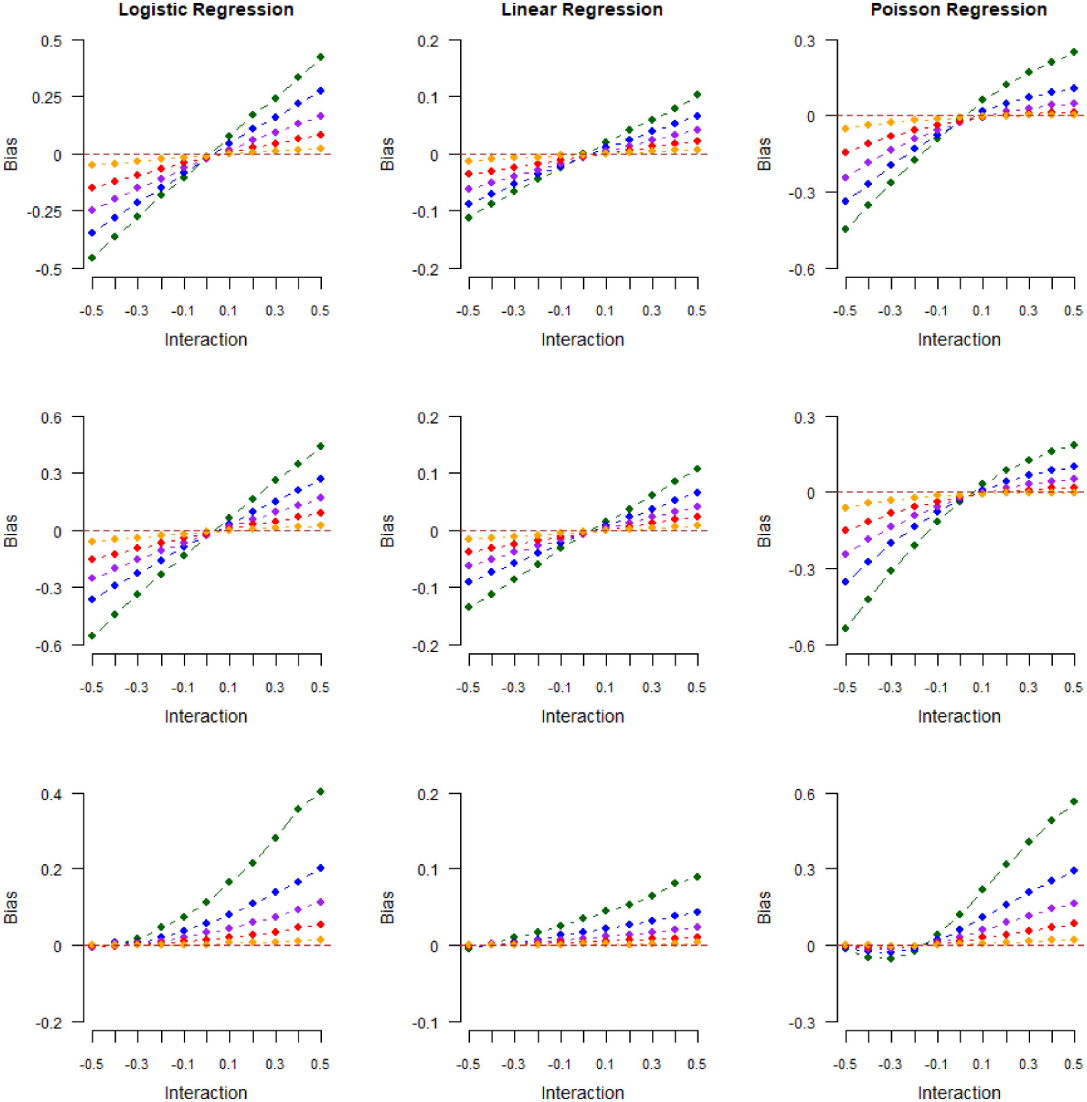
Magnitude of collider bias induced in the exposure–outcome regression coefficient by restricting the analysis to selected ( S=1 ) individuals. Different colors represent different selection probabilities (green: 10% , blue: 30% , purple: 50% , red: 70% , and orange: 90% ). Outcome data were generated from logistic regression (left column), linear regression (middle column), or Poisson regression (right column) and collider values were generated from logistic regression (model $S_{1}$ , top row), probit regression (model $S_{2}$ , middle row), or the “double threshold” model (model $S_{3}$ , bottom row). The bias is plotted against the exposure–outcome interaction $\delta_{3}^{S_{k}}$ in the collider model.

**Figure 5.**
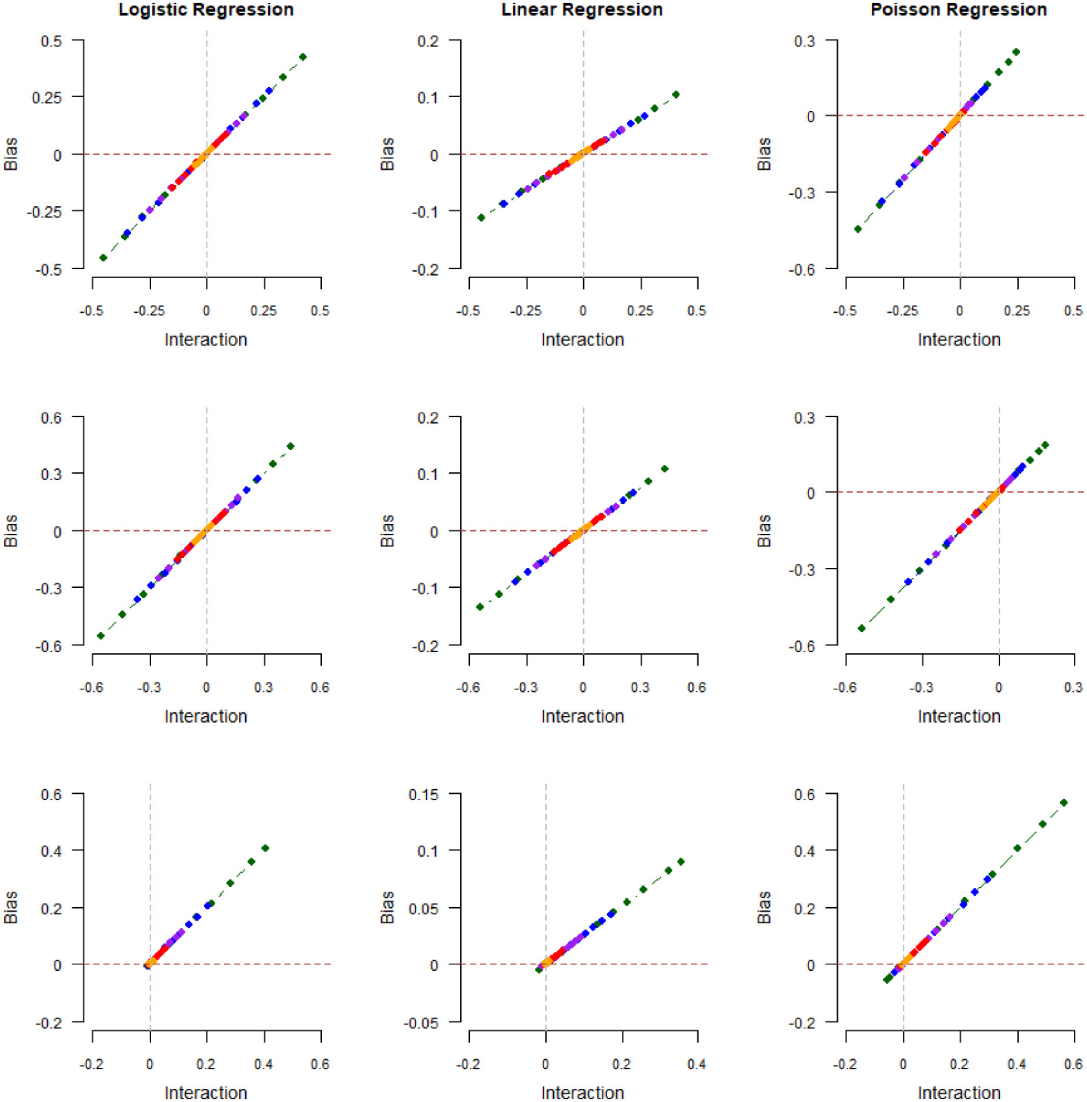
Magnitude of collider bias induced in the exposure–outcome regression coefficient by restricting the analysis to selected ( S=1 ) individuals. Different colors represent different selection probabilities (green: 10% , blue: 30% , purple: 50% , red: 70% , and orange: 90% ). Outcome data were generated from logistic regression (left column), linear regression (middle column), or Poisson regression (right column) and collider values were generated from logistic regression (model $S_{1}$ , top row), probit regression (model $S_{2}$ , middle row) or the “double threshold” model (model $S_{3}$ , bottom row). The bias is plotted against estimated values of the exposure–outcome interaction parameter $\delta_{3}^{S_{0}}$ in a (misspecified) log-additive model for S .

Results from our second numerical experiment are shown in Figures 4 and 5. In Figure 4, we plot the magnitude of collider bias induced in exposure–outcome regression coefficients against the true value of the exposure–outcome interaction in models ( $S_{1}$ )–( $S_{3}$ ), for a range of selection probabilities: 10% (green), 30% (blue), 50% (purple), 70% (red), and 90% (orange). In Figure 5, we do the same for the limiting values ${\tilde{\delta }}_{3}^{S_{0}}$ of the interaction parameter $\delta_{3}^{P}S_{0}$ in the misspecified log-additive model ( $S_{0}$ ). As shown in Figure 4, smaller selection probabilities resulted in more bias across all models considered. However, a smaller proportion of selected individuals also led to a proportional increase in ${\tilde{\delta }}_{3}^{S_{0}}$ values. Hence, in Figure 5, the relationship between bias and interactions was again linear (with a slope of 1 for logistic and Poisson-distributed outcomes and $\sigma^{2}$ for normally distributed outcomes), and the magnitude of collider bias did not depend on the proportion of selected individuals.

In summary, our results suggest that there exist scenarios in which the linear relationship between the magnitude of collider bias and the strength of exposure–outcome interaction on the log-additive scale may hold true even if the collider S is not distributed according to the log-additive model (1). This was shown to be the case for a binary exposure, an outcome distributed according to models ( $Y_{1}$ )–( $Y_{3}$ ) and a collider distributed according to models ( $S_{1}$ )–( $S_{3}$ ). It may be possible to generalize this observation, for example, to models with a non-linear exposure-collider association, but we have not explored this further.

## 4. Real-data application

### 4.1. Data and methods

We also conducted a real-data analysis using data from the Avon Longitudinal Study of Parents and Children (ALSPAC, Boyd et al.^26^ and Fraser et al.^27^). ALSPAC is a longitudinal population-based study that recruited pregnant women residing in Avon, UK, with expected delivery dates between 1 April 1991 and 31 December 1992. The study included 15,447 pregnancies resulting in 15,658 fetuses, 14,901 of which were alive at one year of age. Ethical approval for the study was obtained from the ALSPAC Ethics and Law Committee and the Local Research Ethics Committees. Informed consent for the use of data collected via questionnaires and clinics was obtained from participants following the recommendations of the ALSPAC Ethics and Law Committee at the time. The study website (http://www.bristol.ac.uk/alspac/researchers/our-data/) contains details on all data that is available through a fully searchable data dictionary and variable search tool.

The aim of our analysis is to demonstrate, using real data, the relation between collider bias and exposure–outcome interactions in a log-additive model for selection. To do so, we investigated associations of six maternal traits with sex of offspring at birth (hereafter referred to as offspring sex). These maternal traits included age at delivery, highest educational qualification held, pre-pregnancy body mass index (BMI), depression status, pre-pregnancy smoking, and gestational age. Since offspring sex is determined randomly at conception and unaffected by environmental exposures, one would expect its associations with maternal traits to be null in the absence of bias (perhaps with the exception of gestational age, Divon et al.^28^). We obtained maternal trait and offspring sex data for all ALSPAC families; these data were treated as the “complete sample” for the purposes of our analysis. We also obtained participation data for two follow-up stages of ALSPAC: the “Teen Focus 4” (TF4) clinic visit (age 17+) and the “It’s All About You” (CCU) questionnaire (age 20). These two subsamples were considered as the “selected samples” for our analysis. Selection into the two subsamples differed by offspring sex: in TF4, participation rates were 29.5% for males and 40.0% for females, while in the CCU sample, they were 22.0% for males and 35.9% for females. We explored whether these differences could bias estimated associations between maternal traits and offspring sex in the two selected samples compared to the complete sample.

For each of the six maternal traits, we fitted a logistic regression model with offspring sex as the outcome and the maternal trait as exposure. The models were fitted both in the complete ALSPAC sample and in the two subsamples. We fitted a separate model for each maternal trait to mimic the previous parts of our manuscript, where we only considered one exposure variable. In real-data applications, it may be preferable to conduct a single joint analysis instead, with all six maternal traits included as explanatory variables. Such an analysis is presented in Supplemental Section 6.

We computed regression coefficient estimates from the models regressing offspring sex on each maternal trait, fitted either to the full ALSPAC sample or to the TF4/CCU subsamples. The difference between estimates in the TF4/CCU subsamples and in the full ALSPAC sample was taken as a measure of collider bias for each trait. We then fitted log-additive models for TF4/CCU participation, each time using offspring sex and one of the maternal traits as covariates, and compared the interaction estimates in these models to the magnitude of collider bias. In addition, we fitted logistic regression models for TF4 and CCU participation using offspring sex and one of the six maternal traits as explanatory variables but no interaction terms. Logistic regression is often used to assess which variables associate with study participation, or to adjust for collider bias using inverse probability weighting. We investigated whether parameter estimates from the logistic models could be used to quantify the magnitude of collider bias for each maternal trait.

Note that our application here is conducted for illustrative purposes, to demonstrate the connection between interactions and collider bias in a real dataset. In reality, with access only to the TF4/CCU samples, we would not be able to fit the log-additive model for selection, while with access to the complete ALSPAC sample, there would be no need to restrict the analysis to the TF4/CCU subsamples.

For our analyses, we excluded pregnancies that resulted in miscarriage or early termination ( 3.9% ). We also excluded pregnancies with missing information on maternal traits. Missingness rates for the six maternal traits and sample sizes for our logistic regression analyses are reported in Supplemental Section 5. Missingness in maternal data can be another source of collider bias, and methods such as multiple imputation could be used to adjust for it, under the assumption that the missing data are missing at random (MAR). In this illustrative application, we choose to ignore this source of bias and focus on the bias induced by restricting to participants in the TF4 and CCU subsamples.

### 4.2. Results

Estimated associations between each maternal trait and offspring sex from the respective logistic models are reported in Table 1. We report parameter estimates, standard errors and *p*-values of association between each trait and offspring sex, obtained either from all ALSPAC participants or only from TF4/CCU attendants.

**Table 1.** Associations of maternal traits with offspring sex in ALSPAC, obtained by fitting six separate logistic regression models for offspring sex, each with one maternal trait as the exposure.

	All ALSPAC			TF4			CCU		
Mat trait	${\hat{\beta }}_{1}$	s.e. $\left({\hat{\beta }}_{1}\right)$	*p*-value	${\hat{\beta }}_{1}$	s.e. $\left({\hat{\beta }}_{1}\right)$	*p*-value	${\hat{\beta }}_{1}$	s.e. $\left({\hat{\beta }}_{1}\right)$	*p*-value
Age	0.008	0.003	0.014	0.020	0.006	0.002	0.023	0.007	0.001
Education	−0.018	0.014	0.200	0.067	0.024	0.006	0.116	0.027	$1.6\times 10^{-5}$
BMI	0.004	0.005	0.369	0.010	0.008	0.245	0.007	0.009	0.431
Depression	−0.051	0.053	0.341	−0.039	0.097	0.686	−0.085	0.116	0.461
Smoking	0.054	0.032	0.092	−0.021	0.058	0.720	−0.149	0.067	0.025
Gest age	−0.033	0.007	$3.3\times 10^{-6}$	−0.059	0.016	$1.4\times 10^{-4}$	−0.066	0.017	$1.4\times 10^{-4}$

*Note:* Estimated associations, standard errors and *p*-values computed using data on either all ALSPAC participants, or only those who attended the TF4 visit, or only those who returned the CCU questionnaire. ALSPAC: Avon Longitudinal Study of Parents and Children; CCU: It's All About You; TF4: Teen Focus 4.

Mother’s age at delivery and gestational age were associated with offspring sex in all three samples. The observational association between gestational age and offspring sex has previously been noted in the literature^28^ and could be due to reverse causation, while the association with mother’s age in ALSPAC was fairly weak and could be due to the missing maternal data. Mother’s education was not associated with offspring sex in the full ALSPAC sample but was seen to associate with offspring sex in both TF4 and CCU. Maternal smoking was associated with offspring sex in the CCU sample but not in the TF4 sample or in the full ALSPAC sample, while BMI and depression before pregnancy exhibited no association with offspring sex in any of the three regression analyses. These results suggest collider bias may be affecting the association of maternal education and smoking with offspring sex. This is not unreasonable, as smoking and education are often associated with participation in scientific studies, and at the same time, participation rates in TF4 and CCU differed between males and females, as mentioned earlier.

Table 2 contains the results of fitting log-additive models for TF4 and CCU participation. Again, six different models were fitted, one for each maternal trait. All models also included offspring sex and an interaction between the maternal trait and offspring sex. We report parameter estimates, standard errors and 95% *p*-values for the regression parameters. For comparison, we also report the bias observed in Table 1, computed as the difference between $\beta_{1}$ estimates in the TF4/CCU samples and in the complete sample.

**Table 2.** Parameter estimates, standard errors and *p*-values for a log-additive model of TF4 or CCU participation in terms of offspring sex, maternal traits, and interactions between offspring sex and maternal traits. The observed bias in ${\hat{\beta }}_{1}$ estimates calculated from Table 1 is also reported for comparison.

	Offspring sex			Maternal trait			Interaction			
Variable	Est	StdErr	*p*-value	Est	StdErr	*p*-value	Est	StdErr	*p*-value	Bias
TF4 estimates										
Age	−0.539	0.171	0.002	0.046	0.004	$4.5\times 10^{-33}$	0.007	0.006	0.217	0.012
Education	−0.583	0.086	$1.0\times 10^{-11}$	0.189	0.016	$3.8\times 10^{-33}$	0.083	0.024	0.001	0.085
BMI	−0.399	0.186	0.032	−0.008	0.005	0.149	0.005	0.008	0.562	0.006
Depression	−0.299	0.291	0.304	0.227	0.067	0.001	0.000	0.099	0.998	0.012
Smoking	−0.204	0.086	0.018	−0.364	0.042	$6.5\times 10^{-18}$	−0.084	0.064	0.190	−0.075
Gest age	−0.052	0.519	0.921	0.029	0.009	0.001	−0.007	0.013	0.610	−0.026
CCU Estimates										
Age	−0.767	0.189	$4.8\times 10^{-5}$	0.048	0.004	$3.4\times 10^{-33}$	0.009	0.006	0.176	0.015
Education	−0.937	0.096	$1.7\times 10^{-22}$	0.190	0.016	$3.5\times 10^{-31}$	0.133	0.027	$5.6\times 10^{-7}$	0.134
BMI	−0.526	0.208	0.011	−0.012	0.006	0.031	0.002	0.009	0.807	0.003
Depression	−0.329	0.341	0.335	0.371	0.076	$1.1\times 10^{-6}$	−0.050	0.116	0.665	−0.034
Smoking	−0.196	0.095	0.040	−0.328	0.043	$3.8\times 10^{-14}$	−0.238	0.072	0.001	−0.203
Gest age	−0.014	0.588	0.980	0.041	0.010	$3.8\times 10^{-5}$	−0.012	0.015	0.411	−0.033

Est: estimates; StdErr: standard error; CCU: It's All About You; BMI: body mass index; TF4: Teen Focus 4.

All maternal traits were associated with CCU participation, and all maternal traits apart from BMI were associated with TF4 participation in their respective models. However, evidence of an interaction between the maternal traits and offspring sex was observed only for maternal education (in both samples) and smoking (in the CCU sample). This was in line with our previous analysis, in which maternal education associated with offspring sex among CCU or TF4 participants, and maternal smoking did so among CCU participants. The regression coefficient for the education-offspring sex interaction was estimated to be positive in both log-additive models; this would suggest positive bias. Indeed the TF4 and CCU regression coefficients in Table 1 were both larger than the regression coefficients in the all-ALSPAC analysis (i.e. positive bias). On the other hand, the smoking-offspring sex interaction in the CCU sample was negative, suggesting negative bias, which was indeed the case based on Table 1. Finally, the interaction parameter estimates were a good approximation of the magnitude of bias caused by restricting to the TF4 or CCU subsamples: for all six traits and for both subsamples, a 95% confidence interval for the interaction parameter in the log-additive model contained the observed value of the bias.

The results of fitting the logistic regression models without interactions are given in Table 3. Again, we report parameter estimates, standard errors and *p*-values for offspring sex and each maternal variable, as well as the bias observed in ALSPAC. These results suggest strong associations between all six maternal traits and participation in both samples, with the exception of BMI in the TF4 sample. In addition, participation is also associated with offspring sex, as expected. By fitting the logistic models, an applied researcher could be led to believe that collider bias will occur when studying the associations of maternal traits with offspring sex in TF4/CCU participants. However, as our analysis in Table 1 indicates, bias is present only for maternal education and smoking, and not for the other four maternal traits considered here. This confirms that using a log-additive model with interactions is more informative about collider bias than the commonly used logistic model without interactions.

**Table 3.** Parameter estimates, standard errors and *p*-values for a logistic regression of TF4 and CCU participation on offspring sex and maternal traits.

	Offspring sex			Maternal trait			
Variable	Est	StdErr	*p*-value	Est	StdErr	*p*-value	Bias
TF4 estimates							
Age	−0.524	0.036	$4.6\times 10^{-47}$	0.079	0.004	$8.1\times 10^{-99}$	0.012
Education	−0.515	0.038	$1.9\times 10^{-41}$	0.369	0.016	$1.5\times 10^{-125}$	0.085
BMI	−0.472	0.039	$1.6\times 10^{-34}$	−0.009	0.005	0.069	0.006
Depression	−0.482	0.037	$6.4\times 10^{-38}$	0.345	0.059	$5.7\times 10^{-9}$	0.012
Smoking	−0.494	0.037	$5.0\times 10^{-41}$	−0.603	0.039	$8.3\times 10^{-55}$	−0.075
Gest age	−0.483	0.036	$1.1\times 10^{-41}$	0.038	0.008	$9.8\times 10^{-7}$	−0.026
CCU estimates							
Age	−0.752	0.038	$2.8\times 10^{-85}$	0.078	0.004	$5.0\times 10^{-87}$	0.015
Education	−0.753	0.040	$4.0\times 10^{-79}$	0.371	0.016	$2.2\times 10^{-115}$	0.134
BMI	−0.705	0.040	$3.0\times 10^{-68}$	−0.017	0.005	0.001	0.003
Depression	−0.705	0.039	$5.8\times 10^{-72}$	0.489	0.066	$1.1\times 10^{-13}$	−0.034
Smoking	−0.722	0.039	$9.0\times 10^{-78}$	−0.594	0.041	$1.3\times 10^{-47}$	−0.203
Gest age	−0.704	0.038	$1.1\times 10^{-77}$	0.049	0.009	$9.4\times 10^{-9}$	−0.033

Est: estimates; StdErr: standard error; CCU: It's All About You; BMI: body mass index; TF4: Teen Focus 4.

## 5. Discussion

We have shown that, in three commonly used regression models, the magnitude of collider bias induced in the exposure–outcome association is a linear function of the strength of interaction between the exposure and outcome in a log-additive model for the collider. We have proved these results analytically when the collider is truly distributed according to the log-additive model (1), and explored them via a numerical asymptotic study and a real-data application in cases when the collider does not follow the log-additive model. In Supplemental Tables 1 and 2, we provide a detailed list of all our results, including the distributional assumptions considered for the exposure, outcome, and collider.

Our results can be useful in several ways. As mentioned earlier, modeling selection into a study is an important task for methods that attempt to adjust for collider bias, such as IPW. A key assumption of the IPW method is that the statistical model used to derive weights is correctly specified. If that model is misspecified, the weighted analysis may be biased. There are two aspects to correctly specifying the weighting model: the selection of covariates to be included and the correct specification of the model’s functional form. Advice on the selection of covariates can be found elsewhere; briefly, the variables included in the weighting model should be such that the collider becomes conditionally independent of the exposure and outcome given those covariates.^18,30,29^ Regarding the model’s functional form, it has become common in the literature to implement IPW using logistic regression without interactions as a weighting model; this choice is often made for convenience, and in some applications there is little reason to believe that the logistic model is correctly specified. In fact, a logistic model without interactions offers relatively little flexibility to capture complex relationships between the collider and other variables, and more flexible statistical models should be preferred. This advice has been given in the literature (e.g. Seaman and White^18^) and several authors have investigated the use of flexible statistical and machine learning approaches for the IPW weighting model.^21,20,23,19,22^ Although some applied researchers have utilized these approaches, the use of the logistic model without interactions is still common. Our work contributes to this debate by illustrating the relationship between collider bias and interactions in the collider model, and so emphasizing the limitations of the simple logistic model. From that perspective, if S follows the log-additive model (1), performing IPW with a log-additive weighting model with no interactions is equivalent to assuming that there is no collider bias. In addition, a zero interaction on the logistic scale usually corresponds to a small (but non-zero) interaction on the log-additive scale (in terms of minimizing the Kullback-Leibler divergence between the two models). Therefore, performing IPW with a logistic weighting model with no interactions implicitly assumes that there is only a small degree of collider bias in the analysis. Hence, we recommend that applied researchers using IPW should include interaction terms in the weighting model (logistic or not) to give the method enough flexibility to adjust for collider bias. The same advice will likely hold for other methods that have to model S in order to adjust for the bias.

The fact that collider bias only depends on a single parameter in the simple models considered here can also be useful for sensitivity analyses. In some applications, subject-specific knowledge may allow researchers to assess the strength of exposure–outcome interactions, and hence assess whether collider bias is likely to affect their analyses. In addition, simulation studies are sometimes conducted as a form of sensitivity analysis to explore the impact of collider bias in applications. These simulation studies typically work by varying the associations of the exposure, outcome and other relevant variables with study participation and exploring how much collider bias this induces in analysis results. Our work suggests that it is the interactions (on the log-additive scale) that dictate the magnitude of this bias, and therefore that these interactions should also be varied in addition to (or instead of) the exposure-selection and outcome-selection associations.

In addition, simulation studies are used as a tool for assessing the finite-sample performance of novel methods. Our results may therefore be useful to researchers working on methods to detect or adjust for collider bias. For example, it may be desired to design a simulation where complete-case analysis exhibits a specific degree of bias, and compare that with the performance of a newly developed method. This can be done using a log-additive model with an interaction, and specifying the value of the interaction parameter accordingly.

In some applications, additional information about the causal structure of the association between the exposure, outcome and collider may be available. For example, the effect of X and/or Y on S may be mediated by other known variables. We emphasize that our theory continues to hold in this case. Our results in this manuscript quantify the difference between the conditional (on S ) and unconditional exposure–outcome association. In the presence of collider bias, as shown in Figure 1(a), these two associations will differ. However, collider bias can also occur in applications represented by causal diagrams different to that of Figure 1(a). In such applications, if P(S=1|X,Y) is given by the log-additive model (1), the difference between the conditional and unconditional exposure–outcome associations will still be determined by the parameter $\delta_{3}$ , as our theory suggests. Nevertheless, in some applications, additional information about the causal structure of the exposure-collider and outcome-collider associations may help to assess the plausibility of the log-additive model (1), or to adjust for collider bias. We discuss this in more detail in Supplemental Section 7.

Some extensions of our work are possible. Here, we have focused on three simple statistical models for the exposure–outcome association, namely linear, logistic and Poisson regression. It would be interesting to explore whether similar results hold, for example, in survival analysis models. Another potential extension could be to instrumental variable analyses, which are known to suffer from collider bias.^31,32^

We hope our findings will prove useful to methodologists investigating collider bias, as well as to applied researchers attempting to adjust for the bias in their analyses.

## Supplemental Material

sj-pdf-1-smm-10.1177_09622802241306860 - Supplemental material for Relationship between collider bias and interactions on the log-additive scale Supplemental material, sj-pdf-1-smm-10.1177_09622802241306860 for Relationship between collider bias and interactions on the log-additive scale by Apostolos Gkatzionis, Shaun R Seaman, Rachael A Hughes and Kate Tilling in Statistical Methods in Medical Research

## References

[1] Sperrin M Candlish J Badrick E , et al. Collider bias is only a partial explanation for the obesity paradox. Epidemiology 2016; 27: 525–530. 27075676 10.1097/EDE.0000000000000493 PMC4890843

[2] Viallon V Dufournet M . Can collider bias fully explain the obesity paradox? arXiv:1612.06547v1, 2016.

[3] Hernán MA Hernández-Díaz S Robins JM . A structural approach to selection bias. Epidemiology 2004; 15: 615–625. 15308962 10.1097/01.ede.0000135174.63482.43

[4] Berkson J . Limitations of the application of fourfold table analysis to hospital data. Biometrics 1946; 2: 47–53. 21001024

[5] Mitchell RE Hartley A Walker VM , et al. Strategies to investigate and mitigate collider bias in genetic and Mendelian randomization studies of disease progression. *medRxiv*, 2022. 10.1371/journal.pgen.1010596 PMC9949638 36821633

[6] Greenland S . Response and follow-up bias in cohort studies. Am J Epidemiol 1977; 106: 184–187. 900117 10.1093/oxfordjournals.aje.a112451

[7] Kleinbaum DG Kupper LL Morgenstern H . Epidemiologic research: principles and quantitative methods. New York: John Wiley and Sons, 1982.

[8] Bartlett JW Harel O Carpenter JR . Asymptotically unbiased estimation of exposure odds ratios in complete records logistic regression. Am J Epidemiol 2015, 09; 182: 730–736. 26429998 10.1093/aje/kwv114 PMC4597800

[9] Greenland S . Basic methods for sensitivity analysis of biases. Int J Epidemiol 1996; 25: 1107–1116. 9027513

[10] Greenland S . Bayesian perspectives for epidemiologic research: III. Bias analysis via missing-data methods. Int J Epidemiol 2009, 09; 38: 1662–1673. 19744933 10.1093/ije/dyp278

[11] Nguyen TQ Dafoe A Ogburn EL . The magnitude and direction of collider bias for binary variables. Epidemiol Method 2019, 03; 8: 20170013.

[12] Rothman KJ Lash TL Greenland S . Modern Epidemiology (3rd ed). Philadelphia: Lippincott Williams and Wilkins, 2008.

[13] VanderWeele T . Explanation in Causal Inference: Methods for Mediation and Interaction. New York: Oxford University Press, 2015.

[14] White IR Carlin JB . Bias and efficiency of multiple imputation compared with complete-case analysis for missing covariate values. Stat Med 2010; 29: 2920–2931. 20842622 10.1002/sim.3944

[15] Jiang Z Ding P . The directions of selection bias. Stat Probab Lett 2017; 125: 104–109.

[16] Mansournia MA Nazemipour M Etminan M . Interaction contrasts and collider bias. Am J Epidemiol 2022, 06; 191: 1813–1819. 35689644 10.1093/aje/kwac103

[17] Shahar DJ Shahar E . A theorem at the core of colliding bias. Int J Biostat 2017; 13: 20160055. 10.1515/ijb-2016-0055 28361783

[18] Seaman SR White IR . Review of inverse probability weighting for dealing with missing data. Stat Methods Med Res 2013; 22: 278–295. 21220355 10.1177/0962280210395740

[19] Hill JL . Bayesian nonparametric modeling for causal inference. J Comput Graph Stat 2012; 20: 217–240.

[20] Lee BK Lessler J Stuart EA . Improving propensity score weighting using machine learning . Stat Med 2010; 29: 337–346. 19960510 10.1002/sim.3782 PMC2807890

[21] McCaffrey DF Ridgeway G Morral AR . Propensity score estimation with boosted regression for evaluating causal effects in observational studies . Psychol Methods 2004; 9: 403–425. 15598095 10.1037/1082-989X.9.4.403

[22] Wager S Athey S . Estimation and inference of heterogeneous treatment effects using random forests . J Am Stat Assoc 2004; 113: 1228–1242.

[23] Westreich D Lessler J Funk MJ . Propensity score estimation: neural networks, support vector machines, decision trees (CART), and meta-classifiers as alternatives to logistic regression. J Clin Epidemiol 2010; 63: 826–833. 20630332 10.1016/j.jclinepi.2009.11.020 PMC2907172

[24] Wooldridge JM . Introductory Econometrics: A Modern Approach (5th ed.). Boston: South-Western Cengage Learning, 2012.

[25] Campbell UB Gatto NM Schwartz S . Distributional interaction: Interpretational problems when using incidence odds ratios to assess interaction. Epidemiologic Perspectives and Innovations 2005; 2. https://epiperspectives. biomedcentral.com/articles/10.1186/1742-5573-2-1#citeas 10.1186/1742-5573-2-1 PMC1079911 15745447

[26] Boyd A Golding J Macleod J , et al. Cohort profile: The ‘Children of the 90s’—the index offspring of the Avon Longitudinal Study of Parents and Children. Int J Epidemiol 2013, 04; 42: 111–127. 22507743 10.1093/ije/dys064 PMC3600618

[27] Fraser A Macdonald-Wallis C Tilling K , et al. Cohort profile: The Avon Longitudinal Study of Parents and Children: ALSPAC mothers cohort. Int J Epidemiol 2013, 04; 42: 97–110. 22507742 10.1093/ije/dys066 PMC3600619

[28] Divon MY Ferber A Nisell H , et al. Male gender predisposes to prolongation of pregnancy. General Obstetrics and Gynecology: Fetus-Placenta-Newborn 2002; 187: 1081–1083. 10.1067/mob.2002.126645 12389008

[29] Hernán MA Robins JM . Causal inference: what if. Boca Raton: Chapman & Hall/CRC, 2024.

[30] Howe CJ Cole SJ Lau B , et al. Selection bias due to loss to follow up in cohort studies. Epidemiology 2016; 27: 91–97. 26484424 10.1097/EDE.0000000000000409 PMC5008911

[31] Gkatzionis A Burgess S Conti DV , et al. Bayesian variable selection with a pleiotropic loss function in Mendelian randomization. *bioRxiv*, 2020. 10.1002/sim.9109 PMC8446304 34155684

[32] Hughes RA Davies NM Davey Smith G , et al. Selection bias when estimating average treatment effects using one-sample instrumental variable analysis. Epidemiology 2019; 30: 350–357. 30896457 10.1097/EDE.0000000000000972 PMC6525095

