# Supplementary material for: Relationship between collider bias and interactions on the log-additive scale
Source: Stat Methods Med Res. 2025 Mar 2;34(6):1063–78. doi: 10.1177/09622802241306860 (PMC12209546; doi:10.1177/09622802241306860)
Supplement: sj-pdf-1-smm-10.1177_09622802241306860 - Supplemental material for Relationship between collider bias and interactions on the log-additive scale [file sj-pdf-1-smm-10.1177_09622802241306860.pdf]

Relationship between Collider Bias and Interactions on the Log-Additive Scale

Apostolos Gkatzionis[1], Shaun R. Seaman[3], Rachael A. Hughes[1,2], Kate Tilling[1,2]

[1]MRC Integrative Epidemiology Unit, University of Bristol, UK.
[2]Department of Population Health Sciences, Bristol Medical School, University of Bristol, UK.
[3]MRC Biostatistics Unit, University of Cambridge, Cambridge, UK.

Supplementary Material

1 Summary of all Analyses

Here, we complement the results provided in the main part of our manuscript in several ways. In Section 2 of this supplement, we provide the proofs for the theoretical results stated in our main manuscript; we also provide a brief theoretical exploration of the magnitude of collider bias induced in odds ratio estimates when the collider S follows a logistic distribution. In Section 3 of this supplement, we provide tables of results obtained from our numerical asymptotic study and used to create Figure 2-5 in the main part of the manuscript. In Section 4, we replicate our numerical asymptotic study for the case where the exposure X is normally distributed. In Section 5, we provide missingness rates for the various variables used in our ALSPAC application. In Section 6, we repeat our applied analysis, this time including all six maternal traits in a single regression model. Finally, in Section 7, we discuss the applicability of our results to analyses where there is additional information (in the form of three simple causal diagrams) about the causal relationship between the exposure, outcome and collider.

Tables 1 and 2 provide a summary of the results of all analyses included in our manuscript (both in the main part and in this supplement). Table 1 includes theoretical results; it lists the various modelling assumptions we have considered for the exposure X, outcome Y and collider S and summarizes whether the selection bias induced in the exposure-outcome regression coefficient by conditioning on $S = 1$ is proportional to the strength of interaction between the exposure and the outcome in the S model. We also list the sections of the manuscript containing these results. Table 2 does the same for the results of our numerical asymptotic study.

X	Y	S	Bias $\propto$ Interaction	Section
Any	Logistic	Log-additive	Yes	M2.2.1, S2.1, S2.2
Any	Log-additive	Log-additive	No	M2.2.2, S2.2
Any	Linear	Log-additive	Yes	M2.2.3, S2.3
Any	Poisson	Log-additive	Yes	M2.2.4, S2.4
Any	Logistic	Logistic	No	S2.5

Table 1: A list of all theoretical results included in this manuscript. The columns "X", "Y" and "S" represent the data generating models for the variables X, Y given X and S given X and Y respectively. "Bias $\propto$ Interaction" reports whether the bias induced in the $X - Y$ regression coefficient is proportional to the $X - Y$ interaction in the S model. "Section" lists the sections of the main part (M) and supplement (S) of this manuscript where the corresponding results can be found.

<table>
<tr><th>X</th><th>Y</th><th>S True</th><th>S Fitted</th><th>Bias $\propto$ Interaction</th><th>Section</th><th>Figure</th></tr>
<tr><td>$Ber(0.3)$</td><td rowspan="12">Logistic, Linear or Poisson</td><td>Logistic</td><td>Logistic</td><td>No</td><td>M3.2, S2</td><td>M2, M4</td></tr>
<tr><td>$Ber(0.3)$</td><td>Logistic</td><td>Log-additive</td><td>Yes</td><td>M3.2, S3</td><td>M3, M5</td></tr>
<tr><td>$Ber(0.3)$</td><td>Probit</td><td>Probit</td><td>No</td><td>M3.2, S3</td><td>M2, M4</td></tr>
<tr><td>$Ber(0.3)$</td><td>Probit</td><td>Log-additive</td><td>Yes</td><td>M3.2, S3</td><td>M3, M5</td></tr>
<tr><td>$Ber(0.3)$</td><td>DT</td><td>DT</td><td>No</td><td>M3.2, S3</td><td>M2, M4</td></tr>
<tr><td>$Ber(0.3)$</td><td>DT</td><td>Log-additive</td><td>Yes</td><td>M3.2, S3</td><td>M3, M5</td></tr>
<tr><td>$N(0, 1)$</td><td>Logistic</td><td>Logistic</td><td>No</td><td>S4</td><td>S1</td></tr>
<tr><td>$N(0, 1)$</td><td>Logistic</td><td>Log-additive</td><td>No</td><td>S4</td><td>S2, S3</td></tr>
<tr><td>$N(0, 1)$</td><td>Probit</td><td>Probit</td><td>No</td><td>S4</td><td>S1</td></tr>
<tr><td>$N(0, 1)$</td><td>Probit</td><td>Log-additive</td><td>No</td><td>S4</td><td>S2, S3</td></tr>
<tr><td>$N(0, 1)$</td><td>DT</td><td>DT</td><td>No</td><td>S4</td><td>S1</td></tr>
<tr><td>$N(0, 1)$</td><td>DT</td><td>Log-additive</td><td>No</td><td>S4</td><td>S2, S3</td></tr>
</table>

Table 2: A list of all the results from our numerical asymptotic study. The columns "X" and "Y" represent the data generating models for the variables X and Y given X respectively. "S True" represents the true model from which data for S were generated ("DT" corresponds to the double threshold model), while "S Fitted" represents the model fitted for S given X and Y to estimate the strength of the exposure-outcome interaction (when "S True" and "S Fitted" differ, the latter is misspecified). "Bias $\propto$ Interaction" reports whether the bias induced in the $X - Y$ regression coefficient is proportional to the $X - Y$ interaction in the "S Fitted" model. "Section" lists the sections of the main part (M) and supplement (S) of this manuscript where the corresponding results can be found, and "Figure" lists the figures in which results are plotted.

Note that in addition to these scenarios, sections 2.2.1 and 2.2.2 of the main part of our manuscript also explore in more generality the selection bias in odds ratios and risk ratios, assuming that the outcome is binary but without making any further assumptions about its data generating model.

2 Derivation of Main Results

In this section, we derive mathematically the expressions for collider bias presented in the manuscript. The section follows the same structure as in the main part of the manuscript: we consider in turn

collider bias in odds ratios for binary outcome variables, collider bias in risk ratios for binary outcome variables; collider bias in linear regression coefficients for continuous outcome variables; and collider bias in Poisson regression coefficients for count outcome variables. Throughout this section, we assume that the collider S is distributed according to the log-additive model

$$\log \mathbb{P}(S=1|X,Y) = \delta_0 + \delta_1 X + \delta_2 Y + \delta_3 XY \tag{1}$$

with the parameter δ_3 expressing the exposure-outcome interaction.

2.1 Binary Outcome - Collider Bias on the Odds Ratio Scale

We start with the case of a binary outcome and derive the relationship between the unconditional odds ratio

$$OR_{XY}(x) = \frac{\mathbb{P}(Y=1|X=x+1)}{\mathbb{P}(Y=0|X=x+1)} \times \frac{\mathbb{P}(Y=0|X=x)}{\mathbb{P}(Y=1|X=x)}$$

and the conditional odds ratio

$$OR_{XY|S=1}(x) = \frac{\mathbb{P}(Y=1|X=x+1,S=1)}{\mathbb{P}(Y=0|X=x+1,S=1)} \times \frac{\mathbb{P}(Y=0|X=x,S=1)}{\mathbb{P}(Y=1|X=x,S=1)}$$

We note that

$$\begin{aligned}
\frac{\mathbb{P}(Y=1|X,S=1)}{\mathbb{P}(Y=0|X,S=1)} &= \frac{\mathbb{P}(Y=1|X)}{\mathbb{P}(Y=0|X)} \times \frac{\mathbb{P}(S=1|X,Y=1)}{\mathbb{P}(S=1|X,Y=0)} \\
&= \frac{\mathbb{P}(Y=1|X)}{\mathbb{P}(Y=0|X)} \times \frac{\exp\{\delta_0+\delta_1 X+\delta_2+\delta_3 X\}}{\exp\{\delta_0+\delta_1 X\}} \\
&= \frac{\mathbb{P}(Y=1|X)}{\mathbb{P}(Y=0|X)} \exp\{\delta_2+\delta_3 X\}
\end{aligned}$$

Therefore, the relationship between the conditional and the unconditional odds ratio is given by

$$\begin{aligned}
OR_{XY|S=1}(x) &= \frac{\mathbb{P}(Y=1|X=x+1,S=1)}{\mathbb{P}(Y=0|X=x+1,S=1)} \times \frac{\mathbb{P}(Y=0|X=x,S=1)}{\mathbb{P}(Y=1|X=x,S=1)} \\
&= \frac{\mathbb{P}(Y=1|X=x+1)}{\mathbb{P}(Y=0|X=x+1)} \times \frac{\mathbb{P}(Y=0|X=x)}{\mathbb{P}(Y=1|X=x)} \times \exp\{\delta_2+\delta_3(x+1)-\delta_2-\delta_3 x\} \\
&= OR_{XY}(x)\ \exp\{\delta_3\}
\end{aligned} \tag{2}$$

which is the result reported in the main part of our manuscript. This result has previously been obtained by several authors in the literature, see e.g. Jiang and Ding (2017) and references therein.

The relationship for logistic regression coefficients follows by noting that if

$$\text{logit}\mathbb{P}(Y=1|X) = \beta_0 + \beta_1 X$$

and

$$\text{logit}\mathbb{P}(Y=1|X,S=1) = \beta_0^S + \beta_1^S X$$

the unconditional odds ratio becomes

$$OR_{XY}(x) = \frac{\frac{e^{\beta_0+\beta_1(x+1)}}{1+e^{\beta_0+\beta_1(x+1)}}}{\frac{1}{1+e^{\beta_0+\beta_1(x+1)}}} \times \frac{\frac{1}{1+e^{\beta_0+\beta_1 x}}}{\frac{e^{\beta_0+\beta_1 x}}{1+e^{\beta_0+\beta_1 x}}} = e^{\beta_1}$$

and likewise, the conditional odds ratio becomes $OR_{XY|S=1}(x) = e^{\beta_1^S}$, and then comparing with (2).

The derivation of similar results for multiple exposures and non-linear exposure-collider effects follows using similar arguments.

2.2 Binary Outcome - Collider Bias on the Risk Ratio Scale

We now explore the magnitude of collider bias on the risk ratio scale by comparing the unconditional risk ratio

$$RR_{XY}(x) = \frac{\mathbb{P}(Y=1|X=x+1)}{\mathbb{P}(Y=1|X=x)}$$

to the conditional risk ratio

$$RR_{XY|S=1}(x) = \frac{\mathbb{P}(Y=1|X=x+1, S=1)}{\mathbb{P}(Y=1|X=x, S=1)}$$

We have

$$\begin{aligned}
RR_{XY|S=1}(x) &= \frac{\mathbb{P}(Y=1, S=1|X=x+1) \ / \ \mathbb{P}(S=1|X=x+1)}{\mathbb{P}(Y=1, S=1|X=x) \ / \ \mathbb{P}(S=1|X=x)} \\
&= \frac{\mathbb{P}(Y=1|X=x+1)\,\mathbb{P}(S=1|X=x+1, Y=1)\,\mathbb{P}(S=1|X=x)}{\mathbb{P}(Y=1|X=x)\,\mathbb{P}(S=1|X=x, Y=1)\,\mathbb{P}(S=1|X=x+1)} \\
&= RR_{XY}(x) \times \frac{\mathbb{P}(S=1|X=x+1, Y=1)}{\mathbb{P}(S=1|X=x, Y=1)} \times \frac{\mathbb{P}(S=1|X=x)}{\mathbb{P}(S=1|X=x+1)} \\
&= RR_{XY}(x) \times \frac{\exp\{\delta_0 + \delta_1(x+1) + \delta_2 + \delta_3(x+1)\}}{\exp\{\delta_0 + \delta_1 x + \delta_2 + \delta_3 x\}} \times \frac{\mathbb{P}(S=1|X=x)}{\mathbb{P}(S=1|X=x+1)} \\
&= RR_{XY}(x) \times \exp\{\delta_1 + \delta_3\} \times \frac{\mathbb{P}(S=1|X=x)}{\mathbb{P}(S=1|X=x+1)}
\end{aligned}$$

In addition,

$$\begin{aligned}
\mathbb{P}(S=1|X=x) &= \mathbb{P}(S=1|X=x, Y=1)\,\mathbb{P}(Y=1|X=x) + \\
&\quad + \mathbb{P}(S=1|X=x, Y=0)\,\mathbb{P}(Y=0|X=x) \\
&= \exp\{\delta_0 + \delta_1 x + \delta_2 + \delta_3 x\}\,\mathbb{P}(Y=1|X=x) + \\
&\quad + \exp\{\delta_0 + \delta_1 x\}\,\mathbb{P}(Y=0|X=x)
\end{aligned}$$

Putting everything together, we have

$$\begin{aligned}
RR_{XY|S=1}(x) &= RR_{XY}(x) \times \exp\{\delta_1 + \delta_3\} \times \\
&\quad \times \frac{e^{\delta_0+\delta_1 x+\delta_2+\delta_3 x}\,\mathbb{P}(Y=1|X=x) + e^{\delta_0+\delta_1 x}\,\mathbb{P}(Y=0|X=x)}{e^{\delta_0+\delta_1(x+1)+\delta_2+\delta_3(x+1)}\,\mathbb{P}(Y=1|X=x+1) + e^{\delta_0+\delta_1(x+1)}\,\mathbb{P}(Y=0|X=x+1)} \\
&= RR_{XY}(x) \times \frac{e^{\delta_2+\delta_3(x+1)}\,\mathbb{P}(Y=1|X=x) + e^{\delta_3}\,\mathbb{P}(Y=0|X=x)}{e^{\delta_2+\delta_3(x+1)}\,\mathbb{P}(Y=1|X=x+1) + \mathbb{P}(Y=0|X=x+1)} \qquad (3)
\end{aligned}$$

More specific formulae for the bias on the risk ratio scale can be obtained by making additional modelling assumptions for the exposure-outcome relationship into (3). Consider first a log-binomial regression model for Y,

$$\log\mathbb{P}(Y=1|X=x) = \beta_0 + \beta_1 x$$

The regression coefficient β_1 in this model is known to represent a log-risk ratio:

$$RR_{XY}(x) = \frac{\exp\{\beta_0 + \beta_1 x\}}{\exp\{\beta_0\}} = e^{\beta_1}$$

Moreover, (3) yields a conditional risk ratio of

$$\begin{aligned} RR_{XY|S=1}(x) &= e^{\beta_1} \frac{e^{\delta_2+\delta_3(x+1)}\, e^{\beta_0+\beta_1 x} + e^{\delta_3}\left(1 - e^{\beta_0+\beta_1 x}\right)}{e^{\delta_2+\delta_3(x+1)}\, e^{\beta_0+\beta_1(x+1)} + \left(1 - e^{\beta_0+\beta_1(x+1)}\right)} \\ &= \frac{e^{\delta_2+\delta_3(x+1)+\beta_0+\beta_1(x+1)} + e^{\delta_3+\beta_1}\left(1 - e^{\beta_0+\beta_1 x}\right)}{e^{\delta_2+\delta_3(x+1)+\beta_0+\beta_1(x+1)} + \left(1 - e^{\beta_0+\beta_1(x+1)}\right)} \end{aligned}$$

This means that the (absolute) bias in estimating risk ratios is

$$RR_{XY}(x) - RR_{XY|S=1}(x) = e^{\beta_1}\left(1 - \frac{e^{\delta_2+\delta_3(x+1)+\beta_0+\beta_1 x} + e^{\delta_3}\left(1 - e^{\beta_0+\beta_1 x}\right)}{e^{\delta_2+\delta_3(x+1)+\beta_0+\beta_1(x+1)} + \left(1 - e^{\beta_0+\beta_1(x+1)}\right)}\right) \tag{4}$$

The bias hence depends on both the interaction term δ_3 and the outcome-collider effect parameter δ_2. If $\delta_3 = 0$, (4) becomes

$$RR_{XY}(x) - RR_{XY|S=1}(x) = e^{\beta_1}\left(1 - \frac{e^{\delta_2+\beta_0+\beta_1 x} + \left(1 - e^{\beta_0+\beta_1 x}\right)}{e^{\delta_2+\beta_0+\beta_1(x+1)} + \left(1 - e^{\beta_0+\beta_1(x+1)}\right)}\right)$$

which means that collider bias on the risk ratio scale can arise even in the absence of exposure-outcome interactions. On the other hand, when $\delta_2 = \delta_3 = 0$, we have $RR_{XY}(x) - RR_{XY|S=1}(x) = 0$, meaning that there is no bias on the risk ratio scale. This is expected since the outcome does not associate with S in that case, hence S is not a collider.

Moreover, for a null exposure-outcome association ($\beta_1 = 0$), the risk ratio difference (4) simplifies to

$$RR_{XY}(x) - RR_{XY|S=1}(x) = 1 - \frac{e^{\delta_2+\delta_3(x+1)+\beta_0} + e^{\delta_3}\left(1 - e^{\beta_0}\right)}{e^{\delta_2+\delta_3(x+1)+\beta_0} + \left(1 - e^{\beta_0}\right)}$$

and under both $\beta_1 = 0$ and $\delta_3 = 0$, we obtain again $RR_{XY}(x) - RR_{XY|S=1}(x) = 0$, which means that in the absence of interactions in model (1), collider bias should not affect a null association on the risk ratio scale.

Alternatively, we can investigate the bias on the risk ratio scale under a logistic regression exposure-outcome model,

$$\text{logit}\mathbb{P}(Y = 1|X = x) = \beta_0 + \beta_1 x$$

This is perhaps less relevant in practice because logistic regression coefficients can be interpreted as log-odds ratios, therefore researchers working with logistic regression models tend to use odds ratios to quantify the exposure-outcome association. Nevertheless, if it is desired to perform inference on the risk ratio scale, one can obtain from (3) an unconditional risk ratio of

$$RR_{XY}(x) = \frac{\text{expit}\{\beta_0 + \beta_1 x\}}{\text{expit}\{\beta_0\}} = \frac{e^{\beta_1} + e^{\beta_0+\beta_1(x+1)}}{1 + e^{\beta_0+\beta_1(x+1)}}$$

and a conditional risk ratio of

$$\begin{aligned} RR_{XY|S=1}(x) &= RR_{XY}(x)\,\frac{e^{\delta_2+\delta_3(x+1)}\text{ expit}\{\beta_0+\beta_1 x\}+e^{\delta_3}\,(1-\text{expit}\{\beta_0+\beta_1 x\})}{e^{\delta_2+\delta_3(x+1)}\text{ expit}\{\beta_0+\beta_1(x+1)\}+(1-\text{expit}\{\beta_0+\beta_1(x+1)\})} \\ &= RR_{XY}(x)\,\frac{1+e^{\beta_0+\beta_1(x+1)}}{1+e^{\beta_0+\beta_1 x}}\,\frac{e^{\delta_2+\delta_3(x+1)+\beta_0+\beta_1 x}+e^{\delta_3}}{e^{\delta_2+\delta_3(x+1)+\beta_0+\beta_1(x+1)}+1} \end{aligned}$$

Taking their difference yields a bias of

$$RR_{XY}(x)-RR_{XY|S=1}(x)=1-\frac{1+e^{\beta_0+\beta_1(x+1)}}{1+e^{\beta_0+\beta_1 x}}\,\frac{e^{\delta_2+\delta_3(x+1)+\beta_0+\beta_1 x}+e^{\delta_3}}{e^{\delta_2+\delta_3(x+1)+\beta_0+\beta_1(x+1)}+1} \tag{5}$$

Again, the bias depends on both δ_2 and δ_3. If $\delta_3=0$, the bias is still present and is equal to

$$RR_{XY}(x)-RR_{XY|S=1}(x)=1-\frac{1+e^{\beta_0+\beta_1(x+1)}}{1+e^{\beta_0+\beta_1 x}}\,\frac{1+e^{\delta_2+\beta_0+\beta_1 x}}{1+e^{\delta_2+\beta_0+\beta_1(x+1)}}$$

However, when $\delta_2=\delta_3=0$, the conditional and unconditional risk ratios are equal and the bias is eliminated. Likewise, when $\beta_1=0$, the bias simplifies to

$$RR_{XY}(x)-RR_{XY|S=1}(x)=1-\frac{e^{\delta_2+\delta_3(x+1)+\beta_0}+e^{\delta_3}}{e^{\delta_2+\delta_3(x+1)+\beta_0}+1}$$

and under both $\beta_1=0$ and $\delta_3=0$, the bias is again null.

2.3 Continuous Outcome - Collider bias in Linear Regression Coefficients

We now turn our attention to continuous outcome variables, and assume that the outcome is distributed according to the linear regression model

$$Y=\beta_0+\beta_1 X+\epsilon_Y \quad , \quad \epsilon_Y\sim N(0,\sigma^2)$$

where $\mathbb{E}(Y|X)=\beta_0+\beta_1 X$. Our aim is to explore the bias induced in β_1 by conditioning on $S=1$. To achieve this, we will derive an expression for $\mathbb{E}(Y|X,S=1)$ from first principles. We have

$$\begin{aligned} \mathbb{E}(Y|X=x,S=1) &= \int y f_Y(y|X=x,S=1)dy \\ &= \int y\frac{f_{Y,S}(y,1|X=x)}{f_S(1|X=x)}dy \\ &= \frac{\int y f_{Y,S}(y,1|X=x)dy}{f_S(1|X=x)} \\ &= \frac{\int y f_{Y,S}(y,1|X=x)dy}{\int f_{Y,S}(\psi,1|X=x)d\psi} \\ &= \frac{\int y f_S(1|X=x,Y=y)f_Y(y|X=x)dy}{\int f_S(1|X=x,Y=\psi)f_Y(\psi|X=x)d\psi} \end{aligned}$$

Now use the distributional assumptions for Y, S to get

$$\begin{aligned}
\mathbb{E}(Y|X=x,S=1) &= \frac{\int y \exp\{\delta_0+\delta_1 x+\delta_2 y+\delta_3 xy\} \frac{1}{\sqrt{2\pi\sigma^2}} \exp\left\{-\frac{1}{2\sigma^2}(y-\beta_0-\beta_1 x)^2\right\} dy}{\int \exp\{\delta_0+\delta_1 x+\delta_2 \psi+\delta_3 x\psi\} \frac{1}{\sqrt{2\pi\sigma^2}} \exp\left\{-\frac{1}{2\sigma^2}(\psi-\beta_0-\beta_1 x)^2\right\} d\psi} \\
&= \frac{\int y \exp\left\{\delta_2 y+\delta_3 xy-\frac{1}{2\sigma^2}(y-\beta_0-\beta_1 x)^2\right\} dy}{\int \exp\left\{\delta_2 \psi+\delta_3 x\psi-\frac{1}{2\sigma^2}(\psi-\beta_0-\beta_1 x)^2\right\} d\psi} \qquad (6)
\end{aligned}$$

The integral in the numerator can be computed by completing the square in the exponent:

$$\begin{aligned}
N &= \delta_2 y+\delta_3 xy-\frac{1}{2\sigma^2}(y-\beta_0-\beta_1 x)^2 \\
&= -\frac{1}{2\sigma^2}\left[(y-\beta_0-\beta_1 x)^2-2\sigma^2(\delta_2 y+\delta_3 xy)\right] \\
&= -\frac{1}{2\sigma^2}\left[y^2-2y(\beta_0+\beta_1 x+\sigma^2\delta_2+\sigma^2\delta_3 x)+(\beta_0+\beta_1 x)^2\right] \\
&= -\frac{1}{2\sigma^2}\left[y^2-2yC_1+C_2\right] \\
&= -\frac{1}{2\sigma^2}\left[y^2-2yC_1+C_1^2-C_1^2+C_2\right] \\
&= -\frac{1}{2\sigma^2}\left[(y-C_1)^2\right]+\frac{1}{2\sigma^2}(C_1^2-C_2)
\end{aligned}$$

where $C_1 = C_1(x) = \beta_0 + \beta_1 x + \sigma^2\delta_2 + \sigma^2\delta_3 x$ and $C_2 = C_2(x) = (\beta_0 + \beta_1 x)^2$. Equation (6) then becomes

$$\begin{aligned}
\mathbb{E}(Y|X=x,S=1) &= \frac{\int y \exp\left\{-\frac{1}{2\sigma^2}(y-C_1)^2+\frac{1}{2\sigma^2}(C_1^2-C_2)\right\} dy}{\int \exp\left\{-\frac{1}{2\sigma^2}(\psi-C_1)^2+\frac{1}{2\sigma^2}(C_1^2-C_2)\right\} d\psi} \\
&= \frac{\frac{1}{\sqrt{2\pi\sigma^2}}\int y \exp\left\{-\frac{1}{2\sigma^2}(y-C_1)^2\right\} dy}{\frac{1}{\sqrt{2\pi\sigma^2}}\int \exp\left\{-\frac{1}{2\sigma^2}(\psi-C_1)^2\right\} d\psi}
\end{aligned}$$

The denominator is the integral of a $N(C_1, \sigma^2)$ density across its support, therefore equal to 1, while the numerator is the mean value of such a density, which yields

$$\begin{aligned}
\mathbb{E}(Y|X=x,S=1) &= \frac{1}{\sqrt{2\pi\sigma^2}}\int y \exp\left\{-\frac{1}{2\sigma^2}(y-C_1)^2\right\} dy \\
&= C_1(x) = (\beta_0\delta_2\sigma^2)+(\beta_1+\delta_3\sigma^2)x
\end{aligned}$$

At the same time, if β_0^S, β_1^S denote the regression coefficients of a linear regression model conditional on $S = 1$, we have

$$\mathbb{E}(Y|X=x,S=1) = \beta_0^S + \beta_1^S x$$

and comparing the two expressions for the conditional expectation, we obtain

$$\begin{aligned}
\beta_0^S &= \beta_0+\delta_2\sigma^2 \\
\beta_1^S &= \beta_1+\delta_3\sigma^2
\end{aligned}$$

The proofs for multiple exposure variables and non-linear exposure-outcome associations follow in a similar way.

2.4 Count Outcome - Collider bias in Poisson Regression Coefficients

We now discuss the case of a count outcome variable. Assume that the relationship between X and Y is characterized by the Poisson regression:

$$Y|X = x \sim Poisson(\lambda) \quad , \quad \lambda = \lambda(x) = \exp\{\beta_0 + \beta_1 x\} \tag{7}$$

and that, as earlier, the exposure and the outcome affect S on a log-additive scale. The probability mass function of the Poisson-distributed random variable $Y|X = x$ is:

$$\begin{aligned}
\mathbb{P}(Y = y|X = x) &= \frac{\exp\{-\lambda(x)\}(\lambda(x))^y}{y!} \\
&= \frac{1}{y!}\exp\left\{-e^{\beta_0+\beta_1 x}\right\}\left(e^{\beta_0+\beta_1 x}\right)^y \\
&= \frac{1}{y!}\exp\left\{y(\beta_0 + \beta_1 x) - e^{\beta_0+\beta_1 x}\right\}
\end{aligned}$$

For the conditional model $Y|X = x, S = 1$, we have

$$\begin{aligned}
\mathbb{P}(Y = y|X = x, S = 1) &= \frac{\mathbb{P}(Y = y, S = 1|X = x)}{\mathbb{P}(S = 1|X = x} \\
&= \frac{\mathbb{P}(S = 1|Y = y, X = x)\ \mathbb{P}(Y = y|X = x)}{\sum_\psi \mathbb{P}(S = 1|Y = \psi, X = x)\mathbb{P}(Y = \psi|X = x)} \\
&= \frac{\exp\{\delta_0 + \delta_1 x + \delta_2 y + \delta_3 xy\}\frac{1}{y!}\exp\left\{y(\beta_0 + \beta_1 x) - e^{\beta_0+\beta_1 x}\right\}}{\sum_\psi \exp\{\delta_0 + \delta_1 x + \delta_2 \psi + \delta_3 x\psi\}\frac{1}{\psi!}\exp\left\{\psi(\beta_0 + \beta_1 x) - e^{\beta_0+\beta_1 x}\right\}} \\
&= \frac{\exp\{\delta_2 y + \delta_3 xy\}\frac{1}{y!}\exp\{y(\beta_0 + \beta_1 x)\}}{\sum_\psi \exp\{\delta_2 \psi + \delta_3 x\psi\}\frac{1}{\psi!}\exp\{\psi(\beta_0 + \beta_1 x)\}} \\
&= \frac{\frac{1}{y!}\exp\{y((\delta_2 + \delta_3 x) + (\beta_0 + \beta_1 x))\}}{\sum_\psi \frac{1}{\psi!}\exp\{\psi((\delta_2 + \delta_3 x) + (\beta_0 + \beta_1 x))\}} \\
&= \frac{\frac{1}{y!}\{\exp\{(\delta_2 + \delta_3 x) + (\beta_0 + \beta_1 x)\}\}^y}{\sum_\psi \frac{1}{\psi!}\{\exp\{(\delta_2 + \delta_3 x) + (\beta_0 + \beta_1 x)\}\}^\psi}
\end{aligned}$$

Setting $\kappa(x) = \exp\{(\delta_2 + \delta_3 x) + (\beta_0 + \beta_1 x)\}$, the numerator and denominator can be recognized as probability mass functions of a Poisson distribution with parameter $\kappa(x)$:

$$\begin{aligned}
\mathbb{P}(Y = y|X = x, S = 1) &= \frac{\frac{1}{y!}\{\kappa(x)\}^y}{\sum_\psi \frac{1}{\psi!}\{\kappa(x)\}^\psi} \\
&= \frac{\frac{1}{y!}\exp\{-\kappa(x)\}\{\kappa(x)\}^y}{\sum_\psi \frac{1}{\psi!}\exp\{-\kappa(x)\}\{\kappa(x)\}^\psi} \\
&= \frac{1}{y!}\exp\{-\kappa(x)\}\{\kappa(x)\}^y
\end{aligned}$$

where the denominator is equal to 1 because we are summing over the probability mass function's support. Denoting β_0^S, β_1^S the regression coefficients of a Poisson regression model conditioned on

$S = 1$ and comparing the conditional and unconditional exposure-outcome associations, we have

$$\begin{aligned}
\mathbb{E}(Y|X=x) &= \exp\{\beta_0 + \beta_1 x\} \\
\mathbb{E}(Y|X=x, S=1) &= \exp\left\{\beta_0^S + \beta_1^S x\right\} \\
&= \exp\{(\beta_0 + \delta_2) + (\beta_1 + \delta_3)x\}
\end{aligned}$$

from which it readily follows that $\beta_0^S = \beta_0 + \delta_2$ and $\beta_1^S = \beta_1 + \delta_3$.

The result can be extended to Poisson regression models with multiple exposure variables, or to models with a non-linear exposure-collider association. The proofs for these results are similar to the one given above.

2.5 Collider Bias under a Logistic Model for the Collider

Similar arguments as in the previous sections can be used to obtain expressions for collider bias when the collider S does not follow the log-additive model (1). Here, we provide an indicative example by computing an expression for the collider bias in the logistic regression coefficient β_1 of a binary outcome:

$$\text{logit}\mathbb{P}(Y = 1|X) = \beta_0 + \beta_1 X$$

when the collider also follows a logistic model:

$$\text{logit}\mathbb{P}(S = 1|X, Y) = \delta_0 + \delta_1 X + \delta_2 Y + \delta_3 XY \tag{8}$$

Similar to our proof for collider bias on the odds ratio scale under a log-additive model, we have

$$\begin{aligned}
\frac{\mathbb{P}(Y=1|X,S=1)}{\mathbb{P}(Y=0|X,S=1)} &= \frac{\mathbb{P}(Y=1|X)}{\mathbb{P}(Y=0|X)} \times \frac{\mathbb{P}(S=1|X,Y=1)}{\mathbb{P}(S=1|X,Y=0)} \\
&= \frac{\mathbb{P}(Y=1|X)}{\mathbb{P}(Y=0|X)} \times \frac{\text{expit}\{\delta_0 + \delta_1 X + \delta_2 + \delta_3 X\}}{\text{expit}\{\delta_0 + \delta_1 X\}} \\
&= \frac{\mathbb{P}(Y=1|X)}{\mathbb{P}(Y=0|X)} \times \frac{\frac{\exp\{\delta_0+\delta_1 X+\delta_2+\delta_3 X\}}{1+\exp\{\delta_0+\delta_1 X+\delta_2+\delta_3 X\}}}{\frac{\exp\{\delta_0+\delta_1 X\}}{1+\exp\{\delta_0+\delta_1 X\}}} \\
&= \frac{\mathbb{P}(Y=1|X)}{\mathbb{P}(Y=0|X)} \times \frac{e^{\delta_2+\delta_3 X}\left(1+e^{\delta_0+\delta_1 X}\right)}{1+e^{\delta_0+\delta_1 X+\delta_2+\delta_3 X}}
\end{aligned}$$

Under a logistic regression model for Y, the unconditional odds ratio is $OR_{XY}(x) = e^{\beta_1}$ and the conditional odds ratio is

$$\begin{aligned}
OR_{XY|S=1}(x) &= OR_{XY}(x)\,\frac{\frac{e^{\delta_2+\delta_3(x+1)}\left(1+e^{\delta_0+\delta_1(x+1)}\right)}{1+e^{\delta_0+\delta_1(x+1)+\delta_2+\delta_3(x+1)}}}{\frac{e^{\delta_2+\delta_3 x}\left(1+e^{\delta_0+\delta_1 x}\right)}{1+e^{\delta_0+\delta_1 x+\delta_2+\delta_3 x}}} \\
&= e^{\beta_1}\,e^{\delta_3}\,\frac{\left(1+e^{\delta_0+\delta_1(x+1)}\right)\left(1+e^{\delta_0+\delta_1 x+\delta_2+\delta_3 x}\right)}{\left(1+e^{\delta_0+\delta_1 x}\right)\left(1+e^{\delta_0+\delta_1(x+1)+\delta_2+\delta_3(x+1)}\right)}
\end{aligned}$$

Since regression coefficients in logistic regression models represent log-odds ratios, the magnitude of collider bias in the $X - Y$ regression coefficient is equal to

$$\begin{aligned}\log OR_{XY|S=1}(x) - \log OR_{XY}(x) &= \delta_3 + \log \frac{\left(1 + e^{\delta_0 + \delta_1(x+1)}\right)\left(1 + e^{\delta_0 + \delta_1 x + \delta_2 + \delta_3 x}\right)}{\left(1 + e^{\delta_0 + \delta_1 x}\right)\left(1 + e^{\delta_0 + \delta_1(x+1) + \delta_2 + \delta_3(x+1)}\right)} \\ &= \delta_3 + \log\left(1 + e^{\delta_0 + \delta_1(x+1)}\right) + \log\left(1 + e^{\delta_0 + \delta_1 x + \delta_2 + \delta_3 x}\right) \\ &\quad - \log\left(1 + e^{\delta_0 + \delta_1 x}\right) - \log\left(1 + e^{\delta_0 + \delta_1(x+1) + \delta_2 + \delta_3(x+1)}\right)\end{aligned}$$

Note that expression depends on all four parameters $\delta_0, \delta_1, \delta_2, \delta_3$ in the collider model.

The appeal of the log-additive model (1) over the logistic collider model considered here lies in its simplicity; the fact that collider bias is a linear function of a single parameter, and that this linear relationship holds across a range of outcome models, make the log-additive model a suitable framework for quantifying collider bias in applied studies and designing sensitivity analyses. This would be difficult under the logistic model (8) and even harder for more complex models, for which analytic expressions for the bias may not be available.

3 Tables of Results for the Paper's Numerical Asymptotic Study

In this section we provide numerical values for the results plotted in Section 3 of the main part of the paper. We focus on the first numerical experiment, where 50% of individuals were selected into the study, the results of which were plotted in Figures 2 and 3 of the main part of the paper. For each of the nine scenarios we report the magnitude of collider bias induced in the exposure-outcome association, along with the true value of the exposure-outcome interaction used to generate the data in models (S_1)-(S_3) and the estimated value of the exposure-outcome interaction obtained by fitting the misspecified log-additive model (S_0). These are reported in Table 3. The results confirm the close relationship between collider bias and estimated exposure-outcome interactions on the log-additive scale: the largest difference between the two among our 9 scenarios and 11 interaction values considered in each scenario was 0.003.

4 Continuous Exposure and Misspecification of the Selection Model

So far, we have explored the relationship between collider bias in models (Y_1)-(Y_3) and exposure-outcome interactions in the log-additive model (1) for S. We have shown theoretically that when model (1) is correctly specified, the relationship is linear, and that holds true regardless of the type of the exposure variable. We have also shown numerically that the relationship appears to be linear when model (1) is misspecified and the exposure is a binary variable. However, the same does not hold when the log-additive model is misspecified and the exposure is a continuous variable. In this section, we extend our numerical asymptotic study to demonstrate this fact.

We repeated our first experiment, where 50% of individuals are selected ($S = 1$), but generated exposure values from a $N(0, 1)$ distribution. All nine combinations of outcome models and selection models were considered. Once again, we first plotted collider bias against $X - Y$ interactions on the

$\delta_3^{S_k}$	Binary Outcome		Continuous Outcome		Count Outcome	
	Bias	$\hat{\delta}_3^{S_0}$	Bias	$\sigma^2\ \hat{\delta}_3^{S_0}$	Bias	$\hat{\delta}_3^{S_0}$
Collider Model (S_1) - Logistic Regression						
-0.5	-0.248	-0.248	-0.061	-0.061	-0.243	-0.243
-0.4	-0.197	-0.200	-0.050	-0.050	-0.187	-0.187
-0.3	-0.151	-0.152	-0.039	-0.039	-0.135	-0.137
-0.2	-0.111	-0.110	-0.028	-0.028	-0.092	-0.092
-0.1	-0.063	-0.063	-0.017	-0.017	-0.057	-0.056
0	-0.022	-0.022	-0.007	-0.006	-0.027	-0.026
0.1	0.017	0.016	0.004	0.004	-0.003	-0.003
0.2	0.058	0.057	0.013	0.014	0.015	0.015
0.3	0.095	0.094	0.024	0.023	0.029	0.029
0.4	0.130	0.130	0.033	0.033	0.040	0.039
0.5	0.164	0.164	0.042	0.042	0.048	0.047
Collider Model (S_2) - Probit Regression						
-0.5	-0.250	-0.249	-0.062	-0.062	-0.243	-0.244
-0.4	-0.201	-0.201	-0.050	-0.050	-0.185	-0.185
-0.3	-0.152	-0.151	-0.038	-0.038	-0.138	-0.138
-0.2	-0.108	-0.108	-0.028	-0.028	-0.093	-0.092
-0.1	-0.065	-0.066	-0.016	-0.017	-0.056	-0.056
0	-0.026	-0.025	-0.006	-0.006	-0.025	-0.025
0.1	0.020	0.018	0.003	0.004	-0.001	-0.001
0.2	0.058	0.058	0.014	0.014	0.018	0.018
0.3	0.095	0.096	0.024	0.023	0.032	0.032
0.4	0.130	0.131	0.032	0.033	0.044	0.043
0.5	0.169	0.166	0.042	0.042	0.050	0.050
Collider Model (S_3) - "Double Threshold"						
-0.5	-0.004	-0.003	-0.002	-0.002	-0.005	-0.004
-0.4	0.005	0.001	0.001	0.001	-0.014	-0.013
-0.3	0.005	0.004	0.002	0.002	-0.015	-0.015
-0.2	0.011	0.011	0.005	0.005	-0.006	-0.006
-0.1	0.021	0.019	0.006	0.007	0.011	0.010
0	0.033	0.034	0.009	0.010	0.033	0.034
0.1	0.042	0.043	0.012	0.012	0.060	0.060
0.2	0.060	0.059	0.014	0.015	0.089	0.088
0.3	0.075	0.073	0.017	0.017	0.115	0.115
0.4	0.094	0.094	0.020	0.020	0.143	0.142
0.5	0.113	0.113	0.023	0.024	0.166	0.165

Table 3: Magnitude of collider bias induced in the exposure-outcome regression coefficient by restricting the analysis to selected ($S = 1$) individuals, in the scenario with a binary exposure and an average selection probability of 50%. Outcome data are generated from logistic regression ("Binary Outcome" - left), linear regression ("Continuous Outcome" - middle) or Poisson regression ("Count Outcome" - right) and collider values are generated from models (S_1)-(S_3) as indicated. We report the true value of the exposure-outcome interaction parameter used to generate the data in each of the three collider models ("$\delta_3^{S_k}$"), along with the observed bias in exposure-outcome regression coefficients ("Bias") and estimates of the exposure-outcome interaction parameter in the log-additive model (S_0) ("$\hat{\delta}_3^{S_1}$").

scale of the collider model used to generate the data (logistic, probit or "double-threshold"); these are visualized in Figure 1. The pattern of bias is somewhat different to that in Figure 2 of the main

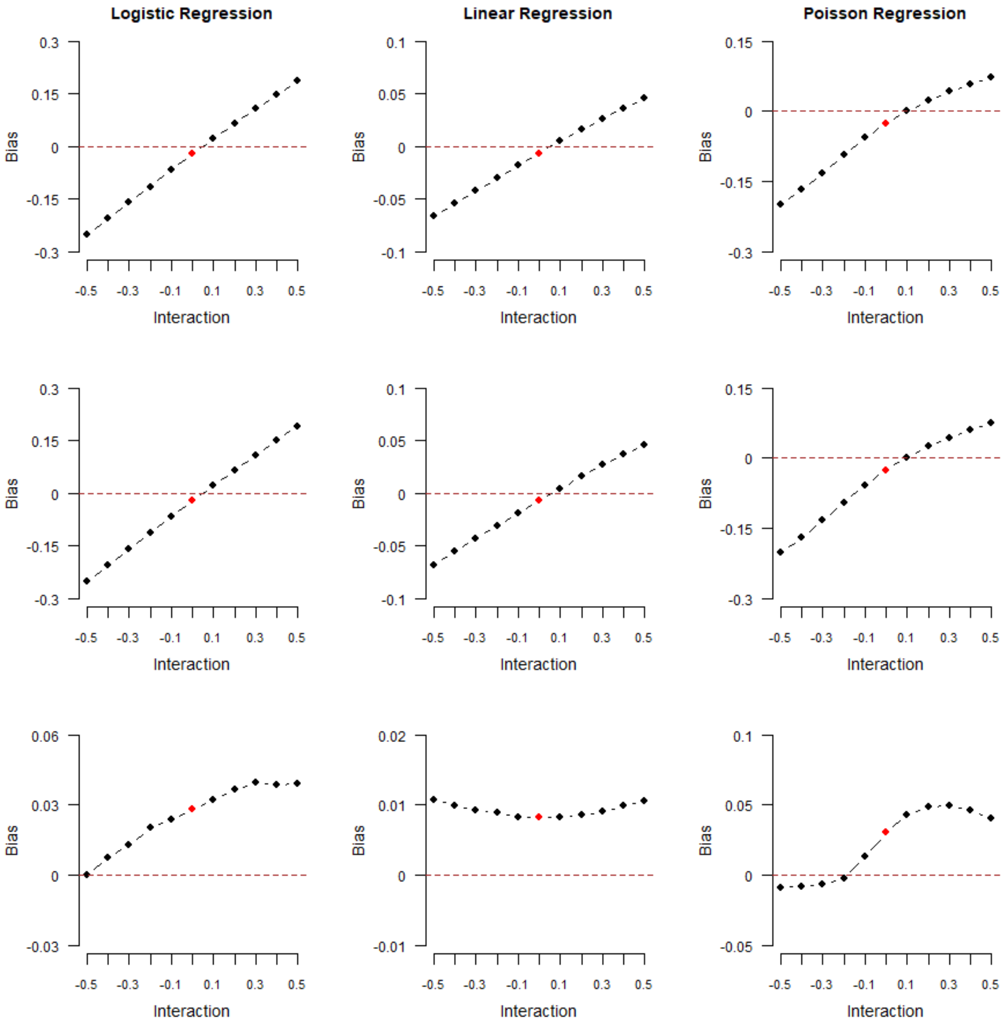

Figure 1: Magnitude of collider bias induced in the exposure-outcome regression coefficient by restricting the analysis to selected ($S = 1$) individuals. Exposure data were generated as a continuous variable ($X \sim N(0, 1)$). Outcome data were generated from logistic regression (left column), linear regression (middle column) or Poisson regression (right column) and collider values were generated from logistic regression (model S_1, top row), probit regression (model S_2, middle row) or the "double threshold" model (model S_3, bottom row). The bias is plotted against the exposure-outcome interaction $\delta_3^{S_k}$ in the collider model. Red color represents the scenario where data were generated with no interaction.

paper for the "double-threshold" model, but otherwise the two figures lead to similar conclusions: the strength of the exposure-outcome interaction affects the magnitude of bias, but their relationship is not easy to characterize.

In Figure 2, we have plotted the bias against estimates $\hat{\delta}_3^{S_0}$ of the $X - Y$ interaction in the log-additive model (S_0). The blue dotted line represents the linear relationship that one would expect to observe based on our theory if the log-additive model was correctly specified. Under misspecification, the relationship between collider bias and $\delta_3^{S_0}$ estimates becomes non-linear; this is more pronounced for the "double-threshold" model but still the case for logistic and probit regression.

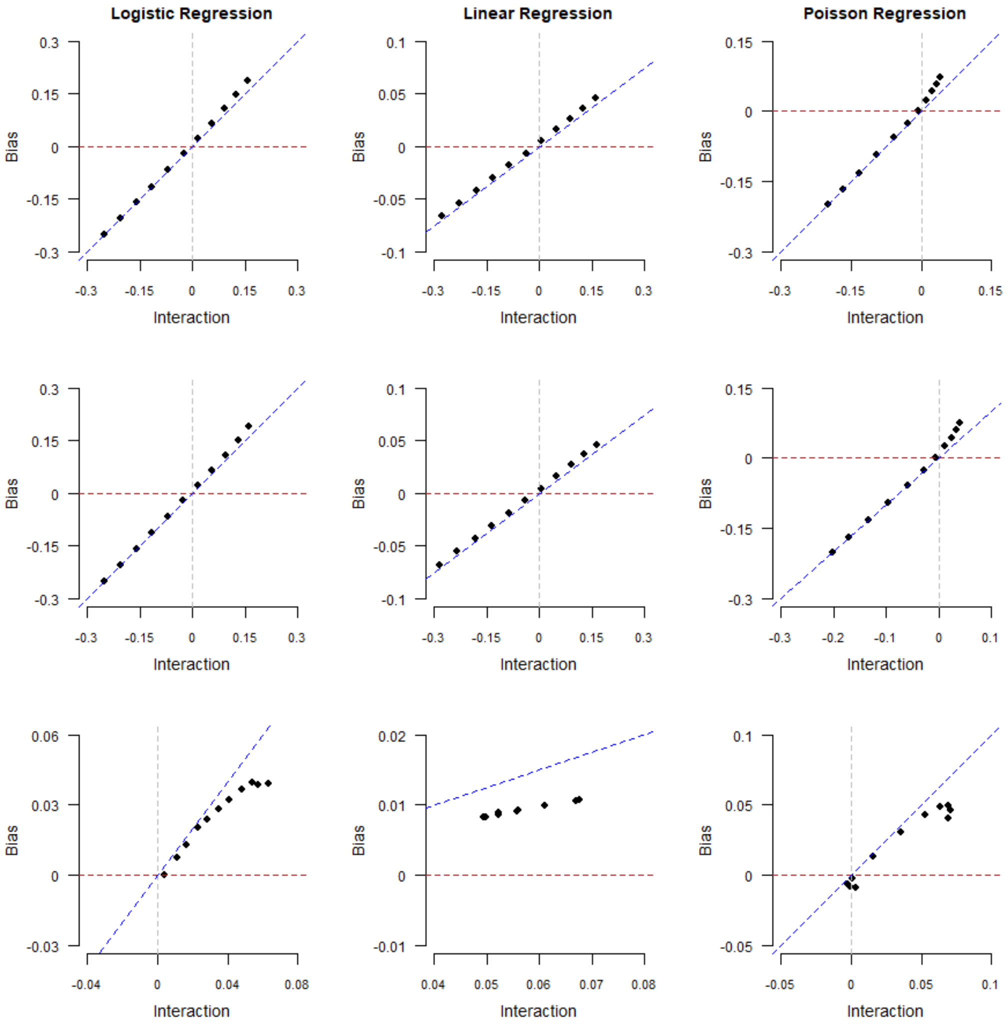

Figure 2: Magnitude of collider bias induced in the exposure-outcome regression coefficient by restricting the analysis to selected ($S = 1$) individuals. Exposure data were generated as a continuous variable ($X \sim N(0, 1)$). Outcome data were generated from logistic regression (left column), linear regression (middle column) or Poisson regression (right column) and collider values were generated from logistic regression (model S_1, top row), probit regression (model S_2, middle row) or the "double threshold" model (model S_3, bottom row). The bias is plotted against the estimated values of the exposure-outcome interaction parameter $\delta_3^{S_0}$ in a (misspecified) log-additive model for S. A gray vertical line represents no interaction ($\hat{\delta}_3^{S_0} = 0$).

The deviation from linearity is relatively small for some combinations of outcome and collider models, so the interaction estimate may still carry some value as an informal assessment of the magnitude of collider bias. Nevertheless, the differences between Figure 2 and Figure 3 of the main paper suggest that, when the collider is not truly distributed according to the log-additive model, the type of the exposure variable matters in determining the bias.

As a consequence of these results, consider a study where the collider S is distributed according

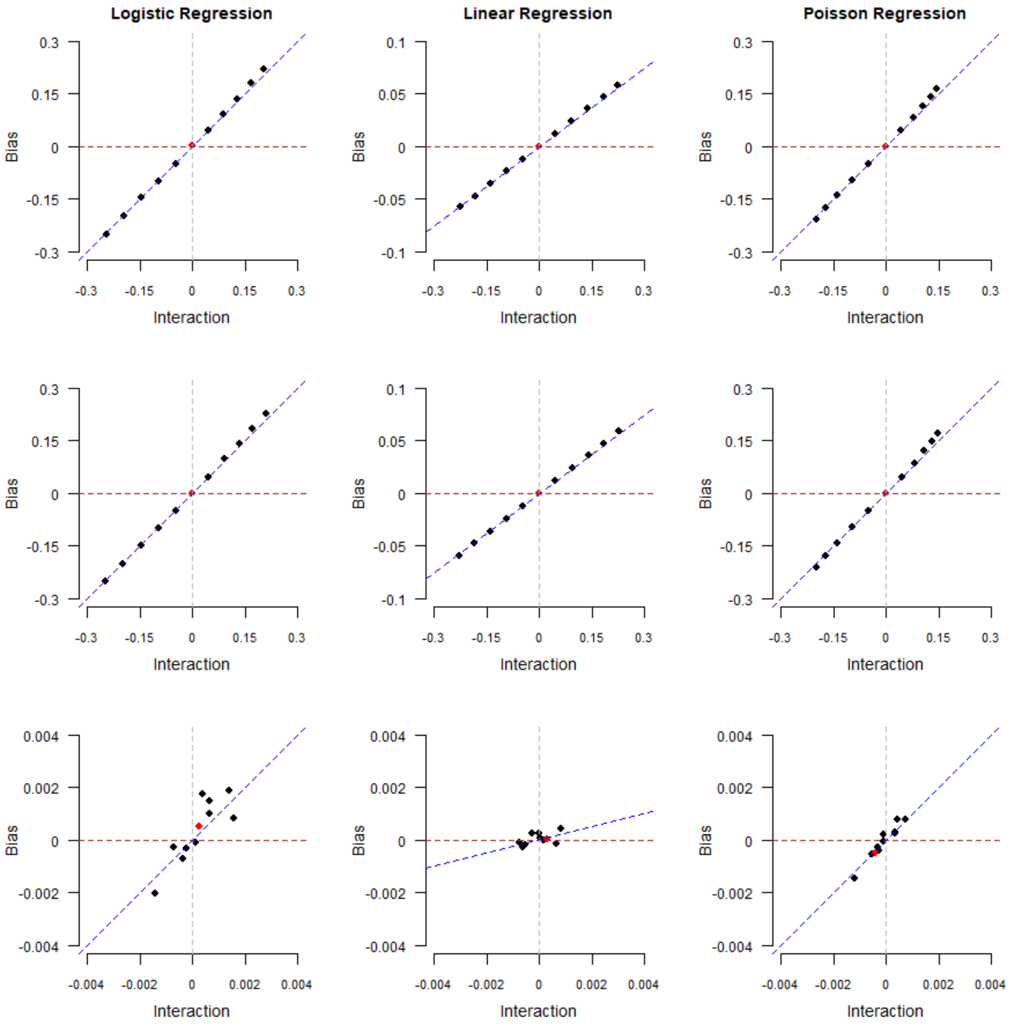

Figure 3: Magnitude of collider bias induced in the exposure-outcome regression coefficient by restricting the analysis to selected ($S = 1$) individuals. Exposure data were generated as a continuous variable ($X \sim N(0,1)$). Outcome data were generated from logistic regression (left column), linear regression (middle column) or Poisson regression (right column) and collider values were generated from logistic regression (model S_1, top row), probit regression (model S_2, middle row) or the "double threshold" model (model S_3, bottom row). The parameters β_1 and δ_2 were set to zero. The bias is plotted against the estimated values of the exposure-outcome interaction parameter $\delta_3^{S_0}$ in a (misspecified) log-additive model for S. Red points represent runs with $\delta_3^{S_k} = 0$, $k = 1, 2, 3$. A grey vertical line represents no interaction ($\hat{\delta}_3^{S_0} = 0$).

to a logistic regression model, and is affected only by the exposure and not by the outcome:

$$\text{logit}\mathbb{P}(S = 1|X, Y) = d_0 + d_1 X \tag{9}$$

The study will not suffer from collider bias, since the absence of a direct $Y - S$ effect means that S s not a collider of the exposure and outcome. However, if one was able to fit the log-additive model (1) for S, the value of the interaction estimate $\hat{\delta}_3$ in that model would be non-zero due to model misspecification. In this case, using heuristic arguments about the plausibility of exposure-collider

Mat Trait	All ALSPAC		TF4		CCU	
	Prop	N	Prop	N	Prop	N
Age	6.5%	978	6.4%	335	4.4%	191
Education	17.1%	2577	9.8%	510	6.9%	301
BMI	22.9%	3437	15.3%	796	12.9%	559
Depression	18.0%	2704	10.6%	554	8.1%	350
Smoking	11.8%	1774	8.4%	436	5.9%	255
Gest Age	6.1%	912	6.4%	335	4.4%	191
Total	—	15038	—	5208	—	4333

Table 4: Missingness rates for each of the six maternal traits and sample sizes used in each of the six logistic regression analyses to estimate the association of offspring sex with the corresponding maternal trait, in the full ALSPAC sample as well as in the two subsamples (TF4 and CCU).

and outcome-collider associations in the context of the study may help to assess the presence of collider bias before attempting to quantify it.

In addition, under model (9), if the (unconditional) exposure-outcome association of interest is also null, this will drive the interaction estimate in the log-additive model towards zero. This is illustrated in Figure 3 where we have repeated the numerical experiment of Figure 2 but set both $\delta_2^{S_k}$ and $\beta_1^{Y_j}$ to zero($k = 1, 2, 3$, $j = 1, 2, 3$). The bias is plotted against estimated interactions in the log-additive model (S_0). In general, these plots are also subject to bias, as in Figure 2. However, in runs where the interaction parameter $\delta_3^{S_k}$ was also set to zero (denoted in red), collider bias does not occur and the interaction estimates in model (S_0) were also close to zero. This was the case for all outcome and all collider models. The pattern of bias may seem less clear for the "double-threshold" model (S_3), but this is due to the scale of the axes: there was very little bias in that model regardless of the value of the interaction $\delta_3^{S_3}$.

5 Missingness Rates for Maternal Traits in ALSPAC

Here, we report the missingness rates for each of the six maternal traits in ALSPAC. The dataset we worked with contained information on 15645 foetuses in ALSPAC. Of those foetuses, 607 had missing offspring sex data, representing miscarriages and early terminations of pregnancy. These were excluded from our analysis, leaving us with 15038 children. In each of the six univariate analyses, we also excluded families with missing values for the corresponding maternal trait. Missingness rates ranged from 6.1% to 22.8% in the full-ALSPAC sample and were slightly smaller in the TF4 and CCU subsamples. These are summarized in Table 4.

6 Joint Analysis for the Real-data Application

Finally, we report results of our real-data application when the associations of the six maternal traits with offspring sex are estimated in a single joint model. Our theory about the connection of collider bias with interactions in log-additive models was presented in terms of models with a

Mat Trait	All ALSPAC			TF4			CCU		
	$\hat{\beta}_1$	s.e. $(\hat{\beta}_1)$	P-value	$\hat{\beta}_1$	s.e. $(\hat{\beta}_1)$	P-value	$\hat{\beta}_1$	s.e. $(\hat{\beta}_1)$	P-value
Age	0.013	0.004	0.002	0.018	0.007	0.011	0.014	0.008	0.083
Education	-0.019	0.016	0.257	0.060	0.028	0.031	0.110	0.031	3.2×10^{-4}
BMI	0.002	0.005	0.692	0.014	0.008	0.911	0.012	0.010	0.221
Depression	-0.075	0.059	0.203	-0.082	0.105	0.433	-0.096	0.125	0.444
Smoking	0.039	0.038	0.306	0.008	0.065	0.896	-0.095	0.075	0.202
Gest Age	-0.054	0.011	3.6×10^{-7}	-0.074	0.018	2.5×10^{-5}	-0.079	0.019	4.6×10^{-5}

Table 5: Associations between each of six maternal traits and offspring sex in ALSPAC, obtained either from all ALSPAC participants, or from those who attended the TF4 visit, or from those who returned the CCU questionnaire.

single exposure variable, hence the choice to model each maternal trait separately in the real-data application in the main part of our manuscript. However, the theory extends straightforwardly to models with multiple explanatory variables, and this is illustrated here.

Again, our objective was to explore the bias induced in associations between six maternal traits and offspring sex, when the analysis was restricted to the subsamples of individuals who attended the TF4 clinic visit or those who completed the CCU questionnaire, compared to the overall ALSPAC sample. To do so, we fitted a single logistic regression model for offspring sex with the six maternal traits as covariates; this was fitted both in the full ALSPAC sample and in the two subsamples. Parameter estimates between the three fits were compared to assess the magnitude of bias that would be induced if the analysis was restricted to the TF4/CCU subsample.

We limited our analysis to families with fully reported maternal data, and also excluded miscarriages and early terminations of pregnancy. This meant that our full-ALSPAC analysis was conducted on a sample of 10689 pregnancies (68.3% of all ALSPAC participants). Of those, 4251 attended the TF4 visit and 3649 completed the CCU questionnaire.

Table 5 contains the results of this analysis. We report parameter estimates, standard errors and p-values of association between each maternal trait and offspring sex, obtained either from all ALSPAC participants or from those who attended TF4 and CCU. Similar to the marginal analyses in the main part of our paper, age at delivery and gestational age were associated with offspring sex (though for age at delivery, the association in the CCU sample was weaker). Education showed no evidence of association with offspring sex in the full ALSPAC sample but did so in the two subsamples, indicating potential collider bias. Unlike the marginal analyses, we did not observe an association of maternal smoking before pregnancy with offspring sex in any of the three logistic regression fits. Finally, maternal BMI and depression did not associate with offspring sex.

To explore whether these associations were due to collider bias, we then fitted a log-additive model for TF4/CCU participation in terms of offspring sex, the six maternal traits and interactions between offspring sex and the maternal traits. We did not include interactions between the maternal traits, as our theory suggests that such interactions play no role in determining collider bias. The interaction parameter estimates from the log-additive model were then compared to the observed bias in ALSPAC. Results are reported in Table 6

All maternal traits apart from BMI were associated with CCU participation, and three maternal traits (education, age at delivery and smoking) were associated with TF4 participation. However only for maternal education was there evidence of an interaction with offspring sex in the log-additive

Mat Trait	TF4				CCU			
	Estimate	Bias in $\hat{\beta}_1$	Std Error	P-value	Estimate	Bias in $\hat{\beta}_1$	Std Error	P-value
Offspring Sex	0.014	—	0.801	0.986	0.419	—	0.895	0.639
Age	0.023	—	0.005	5.0×10^{-7}	0.027	—	0.005	1.8×10^{-8}
Education	0.128	—	0.018	6.8×10^{-13}	0.128	—	0.019	4.3×10^{-12}
BMI	-0.006	—	0.006	0.310	-0.009	—	0.006	0.113
Depression	0.136	—	0.073	0.062	0.238	—	0.080	0.003
Smoking	-0.182	—	0.046	6.5×10^{-5}	-0.141	—	0.047	0.003
Gest Age	0.011	—	0.012	0.363	0.026	—	0.013	0.042
Age x Sex	0.002	0.005	0.007	0.721	-0.003	0.001	0.008	0.716
Education x Sex	0.081	0.079	0.027	0.003	0.131	0.129	0.030	1.4×10^{-5}
BMI x Sex	0.011	0.012	0.008	0.190	0.008	0.010	0.009	0.374
Depression x Sex	-0.030	-0.007	0.106	0.778	-0.058	-0.021	0.123	0.634
Smoking x Sex	-0.034	-0.031	0.070	0.632	-0.140	-0.134	0.078	0.073
Gest Age x Sex	-0.020	-0.020	0.017	0.248	-0.028	-0.025	0.019	0.140

Table 6: Parameter estimates, standard errors and p-values for a log-additive model of TF4 or CCU participation in terms of offspring sex, maternal traits and interactions between offspring sex and maternal traits. The observed bias in $\hat{\beta}_1$ estimates from Table 5 is also reported for comparison.

Mat Trait	TF4			CCU		
	Estimate	Std Error	P-value	Estimate	Std Error	P-value
Offspring Sex	-0.510	0.041	3.8×10^{-35}	-0.747	0.043	9.7×10^{-68}
Age	0.043	0.005	4.2×10^{-21}	0.043	0.005	4.0×10^{-19}
Education	0.283	0.018	1.4×10^{-57}	0.288	0.019	1.4×10^{-54}
BMI	-0.002	0.006	0.702	-0.010	0.006	0.083
Depression	0.194	0.066	0.004	0.318	0.073	1.2×10^{-5}
Smoking	-0.311	0.043	6.1×10^{-13}	-0.286	0.045	2.9×10^{-10}
Gest Age	0.002	0.011	0.829	0.022	0.012	0.067

Table 7: Parameter estimates, standard errors and p-values for a logistic model of TF4 and CCU participation in terms of offspring sex and maternal traits.

model for participation. This was in line with our previous analysis, in which maternal education was the only trait that associated with offspring sex among CCU or TF4 participants but not in the complete ALSPAC sample. In addition, for all maternal traits, interaction parameter estimates were again similar to the observed bias in the ALSPAC analyses, computed from Table 5 as the difference between regression parameter estimates in TF4/CCU samples and in the full ALSPAC sample. A notable difference between bias and interaction parameter estimates was observed for the depression-offspring sex interaction, but the standard errors for the corresponding parameter estimate were larger than for other traits and the difference can likely be attributed to random variation.

As in the main text, we also implemented logistic regression for TF4 and CCU participation using offspring sex and the six maternal traits as explanatory variables but no interaction terms. The results of fitting the logistic regression model to the ALSPAC data are given in Table 7. We report parameter estimates, standard errors and p-values for offspring sex and each maternal variable.

Associations in this joint analysis are generally weaker than in the marginal analyses presented in the main part of our paper, but nevertheless, mother's age at pregnancy, education, depression and smoking before pregnancy all associate with participation into the TF4/CCU samples. As with the marginal analyses, this could lead researchers using the logistic model to worry about collider bias

affecting all four of these traits. However, as Table 5 suggests, only for maternal education is there a cause for concern.

7 Collider Bias in Different Causal Diagrams

This section contains details about the applicability of our results to analyses where some information is known about the nature of the exposure-collider and outcome-collider associations. We start by reiterating that the results presented in the main part of our manuscript do not make explicit assumptions about the causal structure underlying the relationship between the exposure, outcome and collider. We have shown that if the exposure-outcome association is modelled using linear, logistic or Poisson regression and the collider S follows the log-additive model (1), then the magnitude of collider bias induced in the linear, logistic or Poisson regression coefficients by conditioning on $S = 1$ is proportional to the interaction parameter δ_3 in (1). This result continues to hold even if the relationship between S and X, Y is confounded or mediated by other variables, as long as the conditional probability $\mathbb{P}(S = 1|X, Y)$ is given by the log-additive model (1).

Nevertheless, in some applications, additional information about the causal structure of the exposure-collider and outcome-collider associations may help to assess the plausibility of the log-additive model (1), or to adjust for collider bias. Here, we illustrate this for three simple causal diagrams, presented in Figure 4.

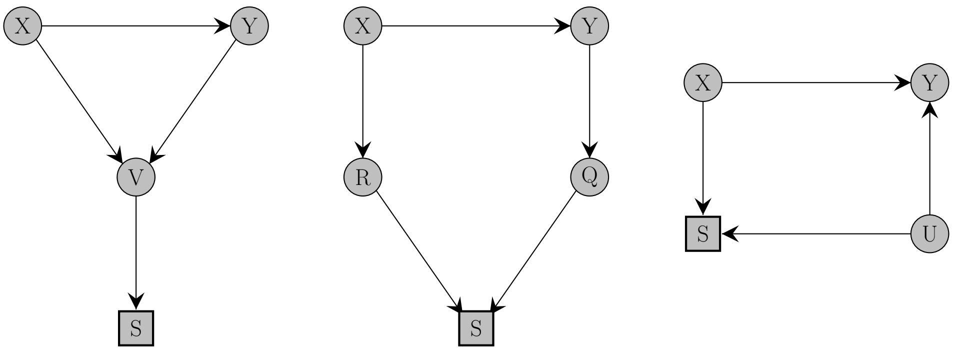

Figure 4: Causal diagrams in which collider bias occurs between the exposure X and the outcome Y.

In diagram 4(a), the exposure and outcome cause the collider through their effects on a third variable V. This could happen, for example, in a cohort study of the effect of body mass index (BMI, X) on smoking (Y), where both smoking and BMI cause cardiovascular disease (V), which causes dropout due to illness (S). For this causal diagram, we will prove that if the $V \sim X, Y$ model is linear regression and the $S \sim V$ model is log-additive, then the $S \sim X, Y$ model is also log-additive. This means that our theory can quantify the magnitude of collider bias in the exposure-outcome association under the above modelling assumptions. This my prove useful in practice, as in some applications it may be easier to elicit modelling assumptions for $V|X = x, Y = y$ and $S|V = v$ separately than for $S|X = x, Y = y$ directly.

Our justification will rely on the extension of our theory about collider bias in linear regression models, when X is vector-valued. We now state this result explicitly. Let $\mathbf{X} = (\mathbf{X_1}, \ldots, \mathbf{X_M})$ be a

vector of random variables and Y an outcome variable that follows the linear regression model

$$Y = \beta_0 + \mathbf{X^T}\beta + \epsilon_\mathbf{Y} \quad , \quad \epsilon_\mathbf{Y} \sim \mathbf{N}(\mathbf{0}, \sigma_\mathbf{Y}^\mathbf{2})$$

where $\beta = (\beta_\mathbf{1}, \ldots, \beta_\mathbf{M})$. In addition, let S be a collider of X and Y distributed according to the log-additive model

$$\log \mathbb{P}(S = 1|\mathbf{X}, \mathbf{Y}) = \delta_\mathbf{0} + \mathbf{X^T}\delta_\mathbf{1} + \delta_\mathbf{2}\mathbf{Y} + \mathbf{X^T}\delta_\mathbf{3}\mathbf{Y} + \mathbf{X^T}\mathbf{\Delta_4}\mathbf{X}$$

where $\delta_\mathbf{1} = (\delta_\mathbf{11}, \ldots, \delta_\mathbf{1M})$, $\delta_\mathbf{3} = (\delta_\mathbf{31}, \ldots, \delta_\mathbf{3M})$ and $\Delta_4 = (\delta_{4jk})$ is a matrix with $\delta_{4jk} = 0$ if $j \geq k$. Then the magnitude of collider bias induced in the regression coefficient β_j, $j = 1, \ldots, M$ by conditioning on $S = 1$ is equal to $\delta_3 j \sigma_Y^2$.

Simply put, when multiple covariates are included in the linear regression model for the outcome, the interaction between each of these covariates and the outcome in a log-additive model for S will determine the collider bias induced in the regression coefficient of that covariate. The bias for covariate X_j only depends on the $X_j - Y$ interaction; it does not depend on $X_k - Y$ interactions for any $k \neq j$, nor does it depend on $X_j - X_k$ interactions. The presence of third or higher-order interactions would also affect the bias but here we only allow for second-order interactions in the model for S.

The proof of this result is very similar to that presented in Section 1.3 for a single covariate and is omitted.

We now return to the causal diagram of Figure 4(a). We assume that the outcome Y is normally distributed according to the linear regression

$$Y = \beta_0 + \beta_1 X + \epsilon_Y \quad , \quad \epsilon_Y \sim N(0, \sigma_Y^2) \tag{10}$$

where X again represents a single covariate. In addition, let V be normally distributed:

$$V = \gamma_0 + \gamma_1 X + \gamma_2 Y + \gamma_3 XY + \epsilon_V \quad , \quad \epsilon_V \sim N(0, \sigma_V^2)$$

and let the collider S follow the log-additive model

$$\log \mathbb{P}(S = 1|V) = \tilde{\delta}_0 + \tilde{\delta}_1 V$$

Substituting V yields

$$\begin{aligned} \log \mathbb{P}(S = 1|X, Y) &= (\tilde{\delta}_0 + \tilde{\delta}_1\gamma_0) + (\tilde{\delta}_1\gamma_1)X + (\tilde{\delta}_1\gamma_2)Y + (\tilde{\delta}_1\gamma_3)XY + \tilde{\delta}_1\epsilon_V \\ &= \delta_0 + \delta_1 X + \delta_2 Y + \delta_3 XY + \tilde{\delta}_1\epsilon_V \end{aligned}$$

and we need to show that the error term ϵ_V does not affect the collider bias. To see this, consider the error term as an additional covariate to be included in the analysis. In the notation of our previous result for vector-valued covariates, we let $\mathbf{X} = (\mathbf{X}, \epsilon_\mathbf{V})$. The "covariate" ϵ_V does not interact with either the exposure or the outcome in its effects on the collider on the log-additive scale. Therefore, in a hypothetical regression model for Y with X and ϵ_V as covariates, collider bias would only occur in the $X - Y$ regression coefficient. The bias would be determined by the $X - Y$ interaction and would be equal to $\delta_3\sigma_Y^2$; ϵ_V will not affect the bias. In addition, due to the collapsibility of linear regression, the $X - Y$ regression coefficient will not change if ϵ_V is removed from the outcome model.

Therefore, the bias induced in the $X-Y$ regression coefficient by conditioning on $S=1$ is equal to $\delta_3\sigma_Y^2$, where $\delta_3=\tilde{\delta}_1\gamma_3$.

Similar arguments can be made for diagram 4(b), For example, this could represent a randomized trial of a smoking cessation programme (X), where those on the programme may have multiple interactions with the programme staff and thus drop out of the study (S) due to research fatigue (R). The outcome (smoking cessation, Y) may cause dropout because those who do not succeed in quitting may feel ashamed (Q) and therefore not wish to return to the study. For this diagram, we show that if $R|X$ and $Q|Y$ are normally distributed and $S|R,Q$ follows a log-additive model, then $S|X,Y$ also follows a log-additive model; therefore, our theoretical results are applicable under these modelling assumptions.

Suppose again that the outcome Y is normally distributed according to the linear regression model (10). Suppose also that the variables R,Q are normally distributed,

$$\begin{aligned} R &= \zeta_0+\zeta_1X+\epsilon_R \quad , \quad \epsilon_R\sim N(0,\sigma_R^2) \\ Q &= \eta_0+\eta_1Y+\epsilon_Q \quad , \quad \epsilon_Q\sim N(0,\sigma_Q^2) \end{aligned}$$

and that the collider S follows the log-additive model

$$\log\mathbb{P}(S=1|R,Q)=\tilde{\delta}_0+\tilde{\delta}_1R+\tilde{\delta}_2Q+\tilde{\delta}_3RQ$$

Substituting R,Q yields

$$\begin{aligned} \log\mathbb{P}(S=1|X,Y) &= \tilde{\delta}_0+\tilde{\delta}_1\zeta_0+\tilde{\delta}_1\zeta_1X+\tilde{\delta}_1\epsilon_R+\tilde{\delta}_2\eta_0+\tilde{\delta}_2\eta_1Y+\tilde{\delta}_2\epsilon_Q \\ &\quad +\tilde{\delta}_3(\zeta_0+\zeta_1X+\epsilon_R)(\eta_0+\eta_1Y+\epsilon_Q) \\ &= (\tilde{\delta}_0+\tilde{\delta}_1\zeta_0+\tilde{\delta}_2\eta_0+\tilde{\delta}_3\zeta_0\eta_0)+(\tilde{\delta}_1\zeta_1+\tilde{\delta}_3\zeta_1\eta_0)X+(\tilde{\delta}_2\eta_1+\tilde{\delta}_3\zeta_0\eta_1)Y \\ &\quad +(\tilde{\delta}_3\zeta_1\eta_1)XY+(\tilde{\delta}_1+\tilde{\delta}_3\eta_0)\epsilon_R+(\tilde{\delta}_2+\tilde{\delta}_3\zeta_0)\epsilon_Q+(\tilde{\delta}_3)\epsilon_R\epsilon_Q \\ &\quad +(\tilde{\delta}_3\zeta_1)\epsilon_QX+(\tilde{\delta}_3\eta_1)\epsilon_RY \\ &= \delta_0+\delta_1X+\delta_2Y+\delta_3XY+\delta_4\epsilon_R+\delta_5\epsilon_Q+\delta_6\epsilon_R\epsilon_Q+\delta_7\epsilon_QX+\delta_8\epsilon_RY \end{aligned}$$

Again, we argue that the error terms ϵ_R,ϵ_Q do not affect the magnitude of bias induced by conditioning on $S=1$. As previously, consider the error terms as additional covariates to be included in the analysis and let $\mathbf{X}=(\mathbf{X},\epsilon_\mathbf{R},\epsilon_\mathbf{Q})$. The interactions between the elements of $\mathbf{X}$ in the collider model will determine the bias in the regression coefficients of a hypothetical linear regression model of Y on $\mathbf{X}$. As there are no three-way interactions, the bias in the $X-Y$ regression coefficient will be fully determined by the $X-Y$ interaction coefficient δ_3; at the same time, δ_8 will determine the bias in the ϵ_R-Y coefficient and the "covariate" interactions ϵ_Q-X and $\epsilon_R-\epsilon_Q$ will not matter. And due to the collapsibility of linear regression, the coefficients will be unchanged if ϵ_R,ϵ_Q are removed from the outcome model. Therefore, the bias in the $X-Y$ regression coefficient will be equal to $\delta_3\sigma_Y^2$, where $\delta_3=\tilde{\delta}_3\zeta_1\eta_1$.

Finally, in diagram 4(c), collider bias is induced between the exposure and a cause U of the outcome. An example for this could be a cohort study of the effect of job stress (X) on cardiovascular disease (CVD, Y), where age (U) increases the chance of CVD and decreases the likelihood of dropout (S). Even if the outcome Y is not a cause of S, the log-additive model (1) could still hold, in which case the interaction parameter δ_3 would still determine the magnitude of collider bias induced. Alternatively if $\mathbb{P}(S=1|X,U)$ follows a log-additive model instead, the $X-U$ interaction in that

model will determine the association induced between X and U due to collider bias. This can then be used along with path rules for causal diagrams (under the assumption that Y is normally distributed) to derive the bias in the exposure-outcome association.

References

Jiang, Z. and P. Ding (2017). The directions of selection bias. *Statistics & Probability Letters 125*, 104–109.
